# The Threat of Potentially Pathogenic Bacteria in the Feces of Bats

**DOI:** 10.1128/spectrum.01802-22

**Published:** 2022-10-26

**Authors:** Yuyuan Huang, Yamin Sun, Qianni Huang, Xianglian Lv, Ji Pu, Wentao Zhu, Shan Lu, Dong Jin, Liyun Liu, Zhengli Shi, Jing Yang, Jianguo Xu

**Affiliations:** a State Key Laboratory of Infectious Disease Prevention and Control, National Institute for Communicable Disease Control and Prevention, Chinese Center for Disease Control and Prevention, Beijing, People’s Republic of China; b Research Institute of Public Health, Nankai University, Tianjin, People’s Republic of China; c Research Center for Functional Genomics and Biochip, Tianjin, People’s Republic of China; d Guangxi Key Laboratory of AIDS Prevention and Treatment, School of Public Health, Guangxi Medical University, Nanning, Guangxi, People’s Republic of China; e Department of Epidemiology, School of Public Health, Shanxi Medical University, Taiyuan, People’s Republic of China; f Research Units of Discovery of Unknown Bacteria and Function, Chinese Academy of Medical Sciences, Beijing, People’s Republic of China; g CAS Key Laboratory of Special Pathogens and Biosafety, Wuhan Institute of Virology, Chinese Academy of Sciences, Wuhan, People’s Republic of China; h Peking University School of Public Health, Beijing, People’s Republic of China; Changchun Veterinary Research Institute

**Keywords:** bat, metataxonomics, culturomics, pathogenic species, fecal microbiota

## Abstract

Bats have attracted global attention because of their zoonotic association with severe acute respiratory syndrome coronavirus (SARS-CoV) and severe acute respiratory syndrome coronavirus 2 (SARS-CoV-2). Previous and ongoing studies have predominantly focused on bat-borne viruses; however, the prevalence or abundance of bat-borne pathogenic bacteria and their potential public health significance have largely been neglected. For the first time, this study used both metataxonomics (16S rRNA marker gene sequencing) and culturomics (traditional culture methods) to systematically evaluate the potential public health significance of bat fecal pathogenic bacteria. To this end, fecal samples were obtained from five bat species across different locations in China, and their microbiota composition was analyzed. The results revealed that the bat microbiome was most commonly dominated by Proteobacteria, while the strictly anaerobic phylum Bacteroidetes occupied 35.3% of the relative abundance in *Rousettus* spp. and 36.3% in *Hipposideros* spp., but less than 2.7% in the other three bat species (*Taphozous* spp., *Rhinolophus* spp., and *Myotis* spp.). We detected 480 species-level phylotypes (SLPs) with PacBio sequencing, including 89 known species, 330 potentially new species, and 61 potentially higher taxa. In addition, a total of 325 species were identified by culturomics, and these were classified into 242 named species and 83 potentially novel species. Of note, 32 of the 89 (36.0%) known species revealed by PacBio sequencing were found to be pathogenic bacteria, and 69 of the 242 (28.5%) known species isolated by culturomics were harmful to people, animals, or plants. Additionally, nearly 40 potential novel species which may be potential bacterial pathogens were identified.

**IMPORTANCE** Bats are one of the most diverse and widely distributed groups of mammals living in close proximity to humans. In recent years, bat-borne viruses and the viral zoonotic diseases associated with bats have been studied in great detail. However, the prevalence and abundance of pathogenic bacteria in bats have been largely ignored. This study used high-throughput sequencing techniques (metataxonomics) in combination with traditional culture methods (culturomics) to analyze the bacterial flora in bat feces from different species of bats in China, revealing that bats are natural hosts of pathogenic bacteria and carry many unknown bacteria. The results of this study can be used as guidance for future investigations of bacterial pathogens in bats.

## INTRODUCTION

Bats (order Chiroptera) are the only flying mammals and have been identified as a natural reservoir of emerging and reemerging infectious pathogens. They are also referred to as mobile “virus banks” (1). Recently, bats have attracted attention globally because of their zoonotic association with severe acute respiratory syndrome coronavirus (SARS-CoV) and severe acute respiratory syndrome coronavirus 2 (SARS-CoV-2) (2). Thus far, prior and current studies have primarily focused on viruses borne by bats, while the prevalence and abundance of pathogenic bacteria in bats and their associated potential public health significance have largely been neglected (3, 4).

Apart from viruses, research has shown that bat feces and intestines contain potential pathogens which can cause serious disease to their hosts or other animals. These potential pathogens include Campylobacter (5, 6), *Clostridium* (7), Salmonella (8–10), *Shigella* (11), and *Bartonella* (12) species. Other bat intestinal and fecal microbes have not shown obvious pathogenicity but may be considered opportunistic pathogens (4), underscoring the importance of exploring the bat microbiota in more detail.

Amplicon sequencing of the 16S rRNA gene (containing nine variable regions, V1 to V9) is the most commonly used method and has proven to be a powerful strategy for the taxonomic classification of bacterial communities (13). The Illumina MiSeq sequencing platform has been used to identify the microbiota at the genus level based on the V3-V4 hypervariable region of the 16S rRNA gene (14). Single-molecule real-time (SMRT) sequencing from Pacific Bioscience (PacBio) has been used to gain a higher taxonomic resolution and identify specific effects (species level) within certain bacterial groups by providing near full-length reads of the 16S rRNA gene (15). Previous studies have indicated that the phyla Firmicutes and Proteobacteria most commonly dominated the bat microbiome, while the phylum Bacteroidetes is relatively rare in the bat microbiome; this is very similar to the microbiome makeup of other flying animals, but distinct from the microbiome compositions of other terrestrial mammals (especially humans and mice) (16, 17). However, no studies have investigated bat fecal microbiota composition through a large-scale culture of bat feces. In addition, the current understanding of the bat fecal microbial community is mainly limited to taxonomic features at the genus level (18).

Herein, we used next-generation sequencing (NGS) for the short-length 16S rRNA gene (V3-V4 region), metataxonomics based on the complete length of the 16S rRNA gene (19), and culturomics (20) to analyze the fecal microbiota of bats collected from five different geographical locations in China to the species level. This study describes the general features of natural bat microbiota at the species level and evaluates the microbiota cytotoxicity and hemolysis in cells while simultaneously analyzing the metabolic features of 16 strains according to their genomes. Numerous pathogenic bacteria with the potential to cause infectious diseases in humans can be found in the fecal microbiota of bats (21).

## RESULTS

### Bat species metadata.

The five species of bats whose feces were sampled in this study come from different habitats (caves in mountains) in southern China, including Yunnan (N25°09′10″, E102°04′39″; N24°33′58″, E102°25′57″), Guangxi (N22°20′54″, E106°49′20″), Chongqing (N30°02′15″, E107°07′4″), and Hubei (N29°46′56″, E114°18′13″) (Fig. S1 in the supplemental material). The temperature of the caves was between 11.5°C and 32.3°C, with the relative humidity ranging from 27.8% to 89.8%. Additionally, the largest number of bats was found in the caves in the Chuxiong Yi Autonomous Prefecture of Yunnan Province, at an altitude of nearly 2,000 m. The BLAST results for the *cytb* gene showed that the bats belonged to the species Rousettus leschenaultii, Taphozous perforates, Hipposideros cervinus, Rhinolophus macrotis, and Myotis scotti, with the percentage of *cytb* gene similarity being between 95.09% and 97.24% (Fig. S1). Furthermore, phylogenetic information deduced from the *cytb* gene also showed that the five bat species were located in separate clusters (Fig. S2).

### The fecal microbiota of bats revealed by metataxonomics and culturomics.

**FIG 1:**
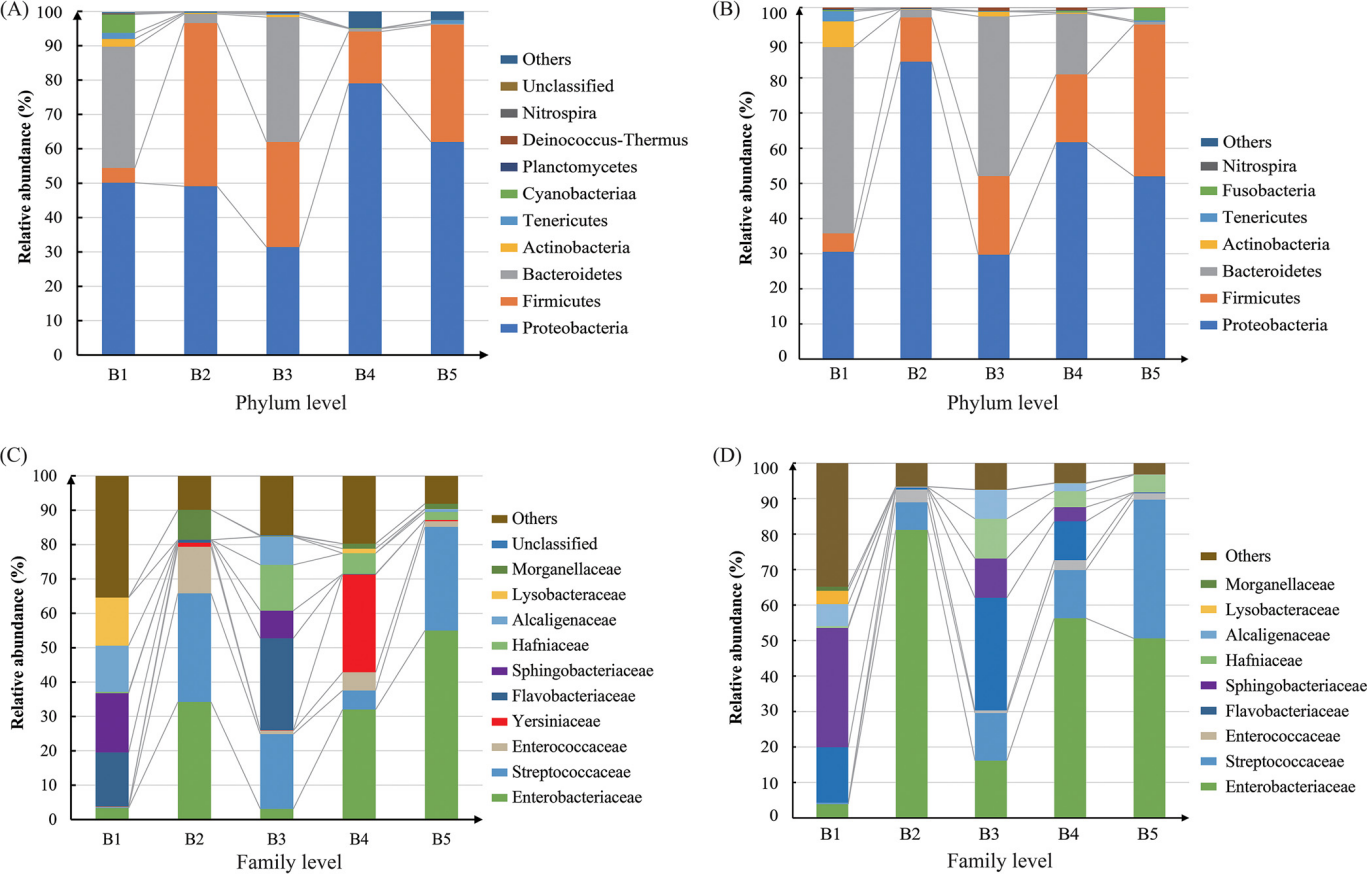
Profile of bat microbiota at the phylum and family levels. Phylum and family levels using the PacBio sequel (A, C) and Illumina HiSeq (B, D) platforms, respectively. B1, *Rousettus* spp.; B2, *Taphozous* spp.; B3, *Hipposideros* spp.; B4, *Rhinolophus* spp.; B5, *Myotis* spp.

The results of the metataxonomics for full-length 16S rRNA gene analysis revealed that Proteobacteria dominated the fecal microbiota of all five bat species, accounting for 50.2%, 49.1%, 31.1%, 79.08%, and 62.0% relative abundance, respectively. Firmicutes, which was also detected in all fecal samples, accounted for approximately one-third or even one-half of the microbial community in *Hipposideros* spp. (B3), *Myotis* spp. (B5), and *Taphozous* spp. (B2) Interestingly, Bacteroidetes occupied 35.3% of *Rousettus* spp. (B1) and 36.3% of *Hipposideros* spp. (B3) and thus can be regarded as dominant. Nevertheless, its proportion in the other three species (B2, B4, and B5) was less than 2.7% (Fig. 1A). According to the Illumina sequencing results, Proteobacteria similarly accounted for the major proportion of microbiota in the bat feces, especially in B2 samples, with an abundance of more than 84.5% (Fig. 1B). More detailed community composition at the family level is shown in Fig. 1C and D. B2 and B5 samples shared a common feature, as they all contained a large proportion of sequences from Enterobacteriaceae and Streptococcaceae. Significantly, Flavobacteriaceae occupied 26.7% in B3 and Yersiniaceae occupied 28.5% in B4 (*Rhinolophus* spp.) samples according to the PacBio sequencing results (Fig. 1C). These two families have been confirmed to include many pathogenic bacteria, such as Flavobacterium meningosepticum (22), Yersinia pestis, Y. pseudotuberculosis, Y. enterocolitica (23) and so on.

**FIG 2:**
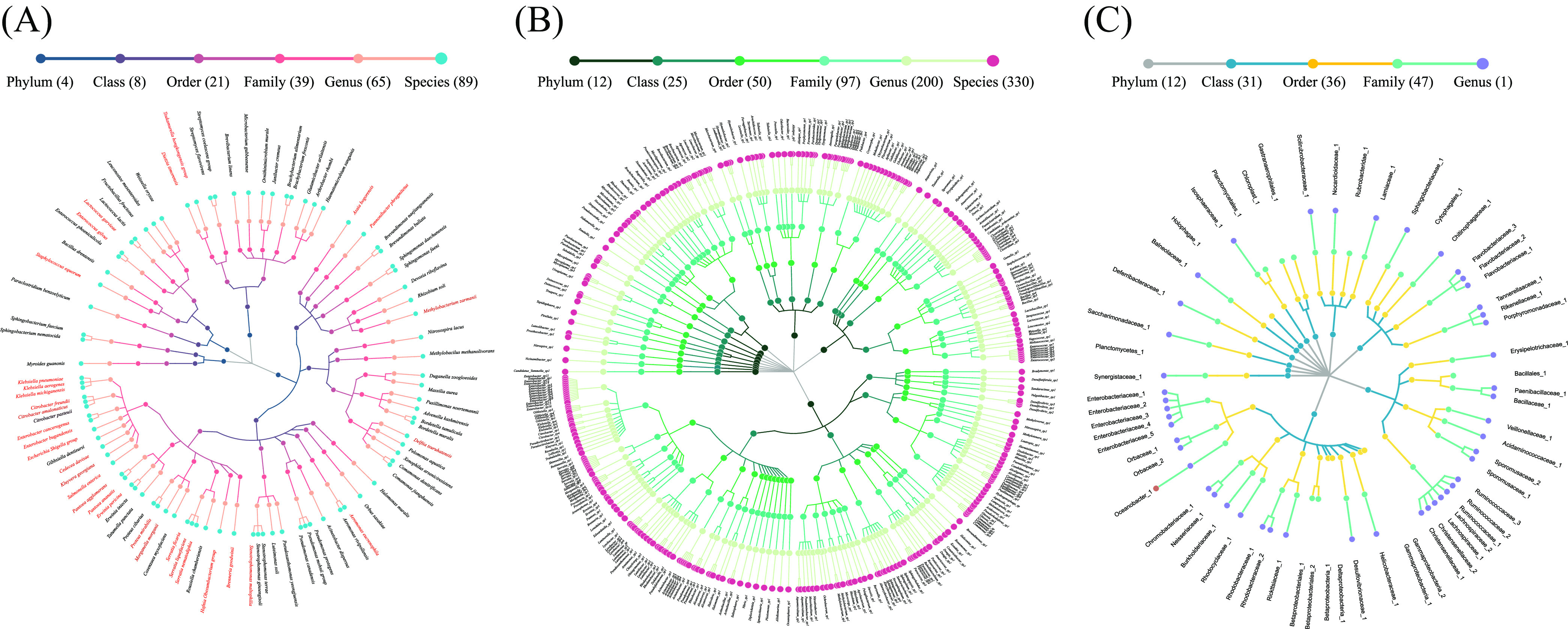
Taxonomic profiles of 480 species-level phylotypes (SLPs) in the gut microbial community of bats. A taxonomic tree of (A) 89 known species (bacteria associated with human, animal, or plant diseases appear in red letters) (27), (B) 330 potentially new species, and (C) 61 potentially higher taxa. Each dot represents an SLP. Descending hierarchical levels are expressed from the inner to the outer rings. The total numbers of SPLs at different hierarchical levels are shown in parentheses.

Furthermore, metataxonomics analysis with almost full-length 16S rRNA gene sequencing generated by the PacBio system allowed precise identification of the microbiome composition up to the species level, especially for unknown taxa (19). After quality filtering and chimera removal, the USEARCH pipeline was used to obtain a total of 12,600 (accession no. PRJNA781098, Table S2) nearly full-length 16S rRNA gene sequences, which were clustered into 1,337 OTUs at 98.7% identity (the boundary for distinguishing between newly discovered and previously known bacterial species) (24). Next, 480 operational phylogenetic units (OPUs; 135 ± 48.08 per sample, Table S2) were detected based on visual inspection of the final *de novo* phylogenetic tree that was generated using the LTP128 (25) or SILVAREF 128 NR databases (26) in order to reconstruct the bat fecal microbiota community, which was ultimately achieved by selecting the most frequent representative sequences within each OTU. The fecal microbiota of the five bat species was identified as 89 known species, 330 potentially novel species, and 61 potentially higher taxa (Fig. 2). The 89 known species were affiliated with 65 genera, 39 families, 21 orders, 8 classes, and 4 phyla, and accounted for 35.2% (4,435 sequences) of the total sequences (Fig. 2A). Taxonomically, 81.5% (391/480) of the members of the bat fecal microbiota comprised unknown bacteria species (Fig. 2B and C). The 330 potentially new species were affiliated with 200 genera, 97 families, 50 orders, 25 classes, and 12 phyla, and accounted for 51.9% (6,544 sequences) of the total sequences (Fig. 2B). The remaining 61 potentially higher taxa included 1 SLP at the genus level, 47 SLPs at the family level, 36 SLPs at the order level, and 43 SLPs at the class or phylum level, and accounted for 12.9% (1,621 sequences) of the total sequences (Fig. 2C).

**FIG 3:**
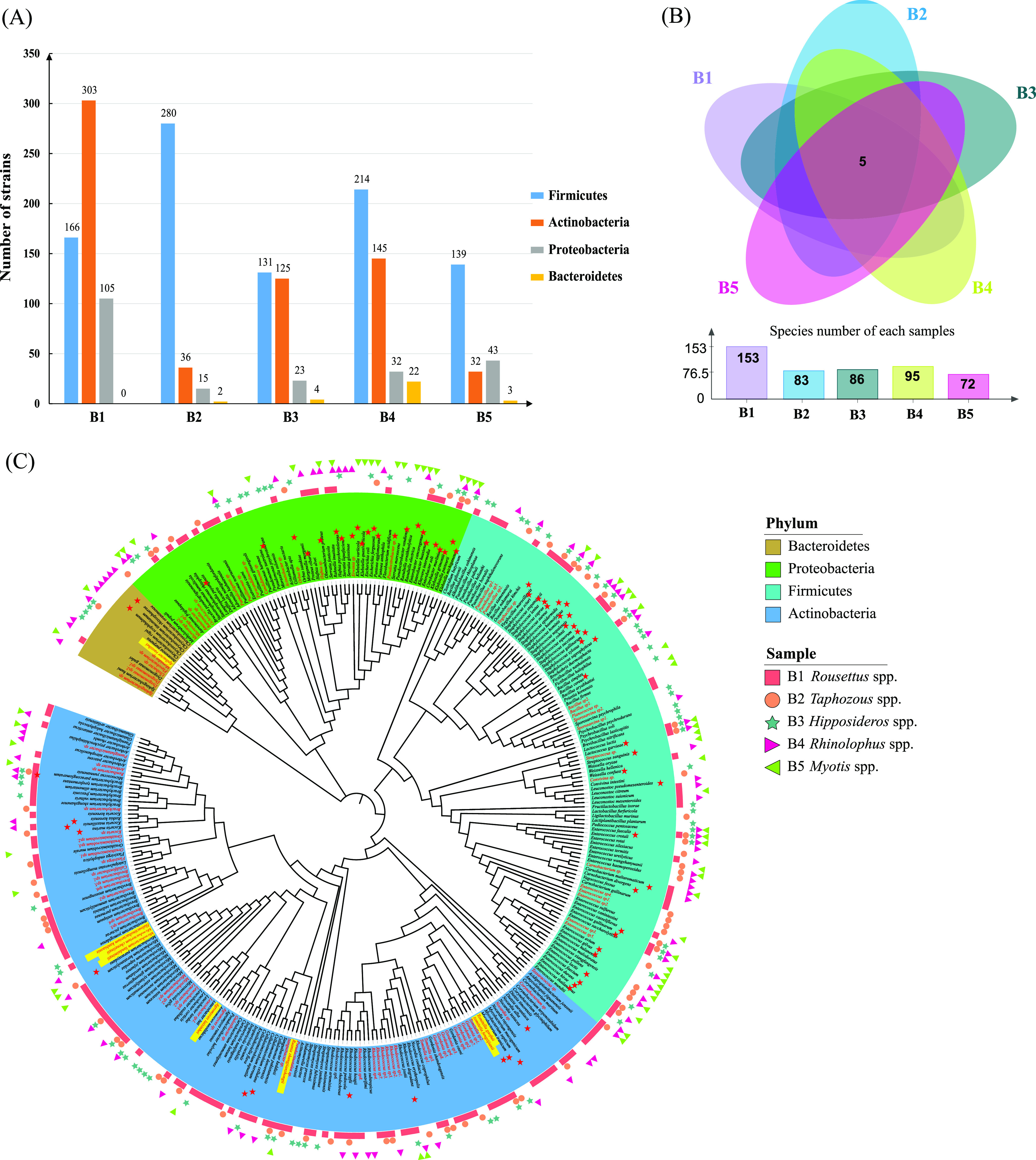
Bacterial diversity in bat feces through culturomics. (A) Number of strains at the phylum level. (B) Number of strains at the species level (325, including 242 known species and 83 putative new species). The number in the Venn diagram represents the species in common between different samples. (C) Cladogram depicting the taxonomic classification of all 325 species. The cladogram is color-coded according to phyla and the outside circle of the graph represents the source of the isolated samples, as shown in the boxes. Red stars indicate bacteria associated with human, animal, or plant diseases:32 Firmicutes, 22 Proteobacteria, 13 Actinobacteria, and 2 Bacteroidetes. Potentially novel species which were described in the present study appear in red (yellow background indicates novel bacterial species which have been validly published).

With the culturomics strategy (Table S1), a total of 1,820 strains were isolated from the fecal samples of the five bat species, and most of the isolates were classified as belonging to the phyla Firmicutes, Actinobacteria, Proteobacteria, and Bacteroidetes (Fig. 3A). On average, approximately 98 species ($\bar{x}\pm s=$ 97.80 ± 31.93) were isolated from each sample, of which 5 (Enterococcus faecalis, E. gallinarum, Klebsiella grimontii, Lactococcus lactis, and L. garvieae) were found in all samples (Fig. 3B). A total of 325 species were identified, and these were classified as 242 known and 83 potentially novel species. In addition, 69 species were identified as those which can potentially cause disease in humans, animals, or plants; of these, 46.4% (27) belonged to Firmicutes, 31.9% (22) belonged to Proteobacteria, and only 18.8% (13) and 2.9% (2) belonged to Actinobacteria and Bacteroidetes, respectively (Fig. 3C). At the species level, 89 and 242 known species were identified by metataxonomics and culturomics, respectively, and a total of 22 species were detected by metataxonomics and culturomics methods (Fig. S3).

### The major microbiota in bat feces.

Of the 480 OPUs detected by metataxonomics, only 4 were shared by all five bat species, including three known species (Enterobacter cancerogenus, Lactococcus lactis, and Citrobacter freundii) and one potentially novel species (*Enterococcus* sp5). A total of 41 OPUs were detected in three-fifths (60% positive rate) of the bat species, including 15 known species, 22 potentially new species, 3 potentially higher taxa, and 1 unclassified group (Fig. S4A). Therefore, 41 of the 480 (8.5%) OPUs were designated high-frequency microbiota, and these were shared by 60% (3/5) of bat species. Of the top 41 most abundant OPUs (to be consistent with the number of species with a 60% positive rate), only 8 (19.5%) were known species, including Advenella kashmirensis, C. freundii, *E. cancerogenus*, Gibbsiella dentisursi, Klebsiella aerogenes, K. pneumoniae, L. lactis, and Morganella morganii. Of these, *E. cancerogenus and* L. lactis were the most prevalent and most abundant species, indicating their potential ecological and biological significance. The four most abundant species (*Lactococcus* sp1, *E. cancerogenus*, K. pneumoniae, and L. lactis) accounted for 30.2% of the total sequences obtained (Fig. S4B).

The multivariate correlation analysis between the top 41 highest-frequency bacteria and the top 41 highest-abundance bacteria across with the metadata showed that most of the bacteria had no statistically significant correlation with the temperature, relative humidity, or altitude of the caves (*P* > 0.05). Interestingly, most of the potentially novel bacteria were positively correlated with feeding habits or the number of bats in the cave (*P* < 0.05) (Fig. S5).

### Pathogenic bacteria revealed by metataxonomics and culturomics.

It must be mentioned that 32 of the 89 (36.0%) known species revealed by metataxonomics were pathogenic species known to cause infection, as evidenced by the published literature (Fig. 2A, Table S3). Moreover, of the 242 known species isolated by means of culturomics, 69 (28.5%) were pathogenic, as also evidenced by the published literatures (Fig. 3C, Table S4).

Among these, Proteobacteria accounted for the highest proportion of all isolates (46.8%, 22/47), followed by Firmicutes (37.6%, 32/85), whereas the lowest proportion of isolates belonged to the phylum Actinobacteria (14.0%, 13/93). Three pathogenic species—namely, E. cancerogenus, C. freundii, and Lactococcus garvieae—were isolated from all five bat species, and the first two species were the top 2 and top 20 most abundant OPUs among all five bat species (Fig. S4B).

Forty-one of the most prevalent OPUs were detected in more than 3 bat species, and 15 (36.6%) were known species. Of these 15 species, 11 (73.3%) were pathogenic, and these included Citrobacter amalonaticus (28), C. freundii, Enterobacter bugandensis, *E. cancerogenus*, Enterococcus gilvus, K. aerogenes, K. pneumoniae, Methylobacterium zatmanii, Morganella morganii (29), Pannonibacter phragmitetus, and Pantoea ananatis (Fig. S4A). Of the 41 most abundant OPUs detected in bats, 8 (19.5%) were named species, 5 of which (62.5%) were pathogenic bacterial species: C. freundii, *E. cancerogenus*, K. pneumoniae, M. morganii, and K. aerogenes (Fig. S4B). Interestingly, K. pneumoniae was detected in 4 of the 5 bat species and was among the top three most abundant species in bat samples tested in this study (30). C. freundii seems to be an important bat microbiome species and was found to be in the top 20 most abundant species (31). *E. cancerogenus* was both the most prevalent and the most abundant fecal bacterium across the five bat species.

### Assessment of the pathogenic potential of the cultured potential new species.

Of the 83 potential new species, 46 (55.4%) were affiliated with 41 genera in which known pathogenic species have been reported (Table S3). According to the phylogenetic tree built in this study, based on the 16S rRNA gene obtained with metataxonomics and culturomics, 28 potentially new species (belonging to 19 genera) were in the same branch or group as known pathogenic bacteria (Fig. S6, blue color: metataxonomics). In addition, it is worth noting that 12 potentially new species belonging to 6 genera (Acinetobacter, *Bartonella*, *Brevibacterium*, *Enterococcus*, *Hafnia*, and *Rhodococcus*) were isolated by culture; phylogenetically, these species were most closely related to known pathogenic species (Fig. S6, green color: culturomics). In particular, *Bartonella sp1.* HY038 and *B. sp2.* HY328 are closely related to *B. tamiae*, which causes acute and chronic infectious diseases (32).

One strain was selected from each isolated species (*n* = 325) to test cytotoxicity using hemolytic activity and lactose dehydrogenase (LDH) cytotoxicity assays. The results revealed that 20 (6.2%) species (>20% hemolytic) were toxic to sheep blood, and 26 (8.0%) species (>20% cytotoxic) were toxic to BV2 cells (Fig. S7). The variations in hemolytic and cytotoxicity assays were stable without abnormal values (Fig. S8). There were 10 strains which had no destructive effect on BV2 cells but had greater than 20% hemolytic activity against sheep red blood cells (RBCs; Fig. S7A, double “^##^”). Twenty-two strains did not show significant hemolytic activity against sheep RBCs but were cytotoxic to BV2 cells (Fig. S7B, single “^#^”). Moreover, 8 species exhibited >20% toxicity toward sheep RBCs and BV2 cells (Fig. S7, single asterisk [*]). Significantly, there were five potential new strains (*Enterococcus sp1.* HY326, *Enterococcus sp2.* HY1045, *Microvirga* sp2. HY445, *Pseudocitrobacter* spp. HY512, and *Rhodococcus* spp. HY359) which may be potentially pathogenic bacterial by being >20% toxic to RBCs or BV2 cells. In particular, Proteus cibarius, Serratia fonticola, Serratia liquefaciens, and Staphylococcus aureus exhibited greater than 50% toxicity (Fig. S9).

**FIG 4:**
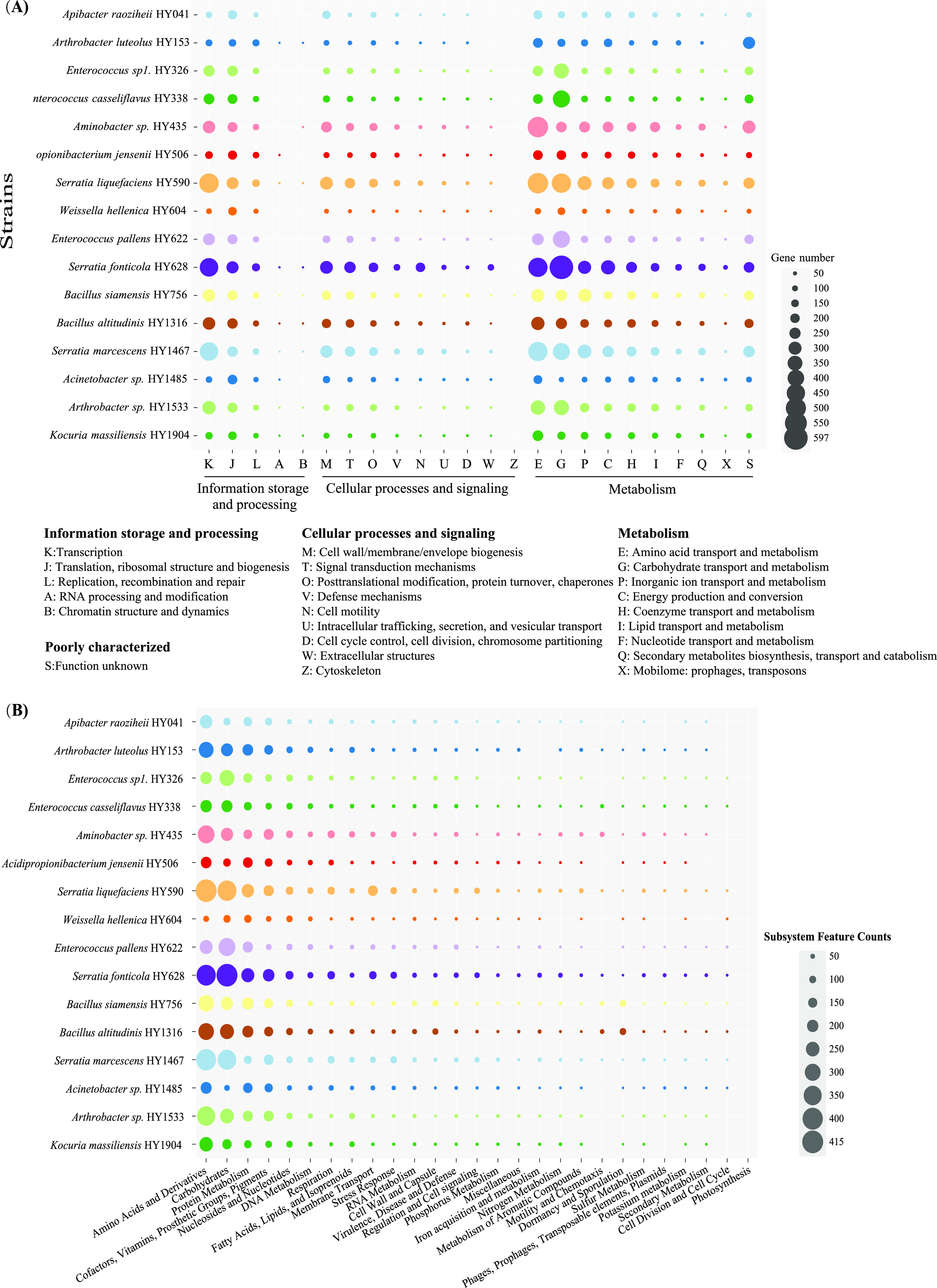
Distributions of the Clusters of Orthologous Groups (COGs) (A) and subsystem categories (B) in the 16 sequenced genomes isolated from the bat feces. The COGs and subsystems are bubble coded, and bubble size indicates the number of genes.

Hemolytic and cytotoxic genes were extracted from 16 bacterial genomes based on the gene annotation and functional prediction, and these included offensive virulence factors and hemolysin genes (Table S5). The statistical results showed that hemolysis and cytotoxicity were positively correlated and that they were both proportional to adherence genes (*P* < 0.005). Additionally, the secretion system genes were also positively correlated with the cytotoxicity of strains (*P* < 0.005), but there was no statistically significant correlation with hemolysis. Of note, the number of toxin genes and hemolysin genes was not significantly different from the cytotoxicity and hemolytic results (Fig. S10). Moreover, the Clusters of Orthologous Groups (COG) database was used for the classification of the genes in the sequenced genomes (Fig. 4A). The results revealed that the highest numbers of genes contained in these genomes were associated with transcription (COG-K), translation (COG-J), and DNA replication and repair (COG-L) for information storage and processing. For cellular processes and signaling, the genes involved in cell wall/membrane/envelope biogenesis (COG-M) and signal transduction mechanisms (COG-T) were commonly abundant in the sequenced genomes. Based on our analysis of the genes associated with metabolism, we found that the genes in the bacteria in bat feces were involved in the preference of carbon sources composed of amino acids (COG-E) and in carbohydrate transport and metabolism (COG-G), which was aligned with the subsystem feature counts shown in Fig. 4B. We observed that the functions of inorganic ion transport and metabolism (COG-P), energy production and conversion (COG-C), and protein metabolism were also abundant in the bacterial genomes. However, the proportions of genes involved in secondary metabolism, potassium metabolism, and sulfur metabolism were relatively small. Notably, a large quantity of the genes in these bacterial genomes were poorly characterized, and their functions remain to be identified (COG-S) (Fig. 4).

### Isolation origins and oxygen tolerance of the bat fecal microbiota.

Detailed information on the known species detected by metataxonomics or isolated by culturomics was obtained from the LPSN (List of Prokaryotic names with Standing in Nomenclature, https://www.bacterio.net/species) and BacDive (https://bacdive.dsmz.de/). It was found that 71.9% and 71.5% of the aerobic bacteria detected by metataxonomics and culturomics, respectively, were originally from environmental sources, clinical specimens, food, insects, and so on. The facultatively anaerobic or anaerobic species could also be isolated from various sources (Fig. S11).

## DISCUSSION

Compared to the number of studies on bat viruses that have drawn much research attention over the past few decades, knowledge about bat bacteria seems to be lagging behind. Because of the wide array of uncultivable microorganisms, studies relying only on traditional culture methods were obviously limited. Similarly, sequencing analysis alone without isolation and culture cannot provide a comprehensive understanding of strains (16). In addition, current understanding of the bat fecal microbial community is mainly limited to taxonomic features at the genus level or higher. For example, Vengust reported that five genera from the phylum Proteobacteria accounted for 50% of sequences (18). In another study, a heatmap constructed from data on 30 major genera showed that *Lactococcus* and *Enterococcus* were consistently detected in all samples and belonged to the top three abundances (16), which was similar to the findings of our study; however, we were able to obtain results at the species level (*Lactococcus* spp., L. lactis, and *Enterococcus* spp.). Furthermore, a total of 35 bacterial species belonging to different genera were isolated from the bat gut by the conventional plating method as described by Selvin (27), and a variety of pathogenic bacteria or novel species of bacteria could also be isolated from bat feces (33–36); however, it is rare to obtain pure strains from bat feces through large-scale isolation with different media, atmospheres, and temperatures (Table S1). Notably, this is the first study to analyze the bat fecal microbial community at the species level by combining metataxonomics with culturomics.

Proteobacteria was the dominant phylum in the microbiomes of the five bat species (Fig. 1A and Fig. 3C). The presence of Proteobacteria is well recognized as a possible microbial signature of pathogenicity (37). However, strictly anaerobic bacteria from phylum Bacteroidetes were almost absent in the bat species. This phenomenon was similar to that reported in birds (*Ovipara*) but different from that seen in other mammals (16, 17). One reason for this may be that the intestinal lengths of bats and birds are shorter than those in mice and other mammals to facilitate more efficient powered flight by reducing intestinal content retention times and decreasing mass (38–40). Additionally, shorter intestines also do not provide a suitable anaerobic environment for anaerobic bacteria such as those from the phylum Bacteroidetes (17). In addition, the most likely reason is that the feces were exposed to the air of the caves for some time, resulting in the death of some strictly anaerobic bacteria, reducing the probability of detection and culture. Interestingly, Proteobacteria has also been reported as the most abundant phylum in the airborne microbiome (41), suggesting that there is a positive correlation between the intestinal flora of flying animals and the air to which flighted animals are more constantly exposed as a source of oxygen.

It should be mentioned that the air microbiome in the cave might have the potential to contaminate the fecal microbiota of the bats living in the same cave. However, we mixed all samples in a given cave into one pooled sample. The potential contamination effect of the air microbiome should have been significantly diminished for the cave-specific sample and would have no effect on the samples from other caves. Indeed, the high frequency and abundance of bacteria in bats were independent of temperature, relative humidity, and altitude according to the statistical analysis results (Fig. S5).

Moreover, it is well known that intestinal bacterial diversity is influenced by various factors, including host diet, age, phylogeny, and gut type (42–44). In particular, host diet emerges as a pivotal determinant of gut microbiota community structure and function (45). Based on diet, bats can be divided into frugivorous, nectarivorous, insectivorous, and sanguivorous (46). In our study, five fecal samples were obtained from bats belonging to the genera *Rousettus*, *Taphozous*, *Hipposideros*, *Rhinolophus*, and *Myotis*, which can be mainly divided into fruit bats (*Rousettus* spp.) and insectivorous bats (*Taphozous* spp., *Hipposideros* spp., *Rhinolophus* spp., *and Myotis* spp.). Metataxonomics revealed that only three known microbial species were shared by all five bat species. Two of these were pathogenic and can cause brain abscesses associated with conditions such as meningitis, osteomyelitis, and pneumonia (47–49). These species were E. cancerogenus and C. freundii, which have been detected or isolated from *Dendrolimu kikuchii* and pollen (50, 51), *Hyalessa maculaticollis*, Galleria mellonella (52, 53), and leafhoppers, respectively. The materials in which the two pathogenic species have been detected or isolated can be used as a food source for bats. Interestingly, some potential novel species may be mostly related to feeding habits or bat numbers (Fig. S5), which can explain why we could isolate so many novel bacteria from the feces of *Rousettus* spp. (Fig. 3C) and how the pathogenic bacteria in bat feces might be obtained from the food they ingest.

Significantly, a novel species of the genus *Lactococcus* was the most abundant in bats, and L. lactis was a common to all five bat species. L. lactis not only contributes to digestion but also generates a proton motive force during citrate metabolism (54). In addition, L. lactis can regulate intestinal immunity and help suppress pathogen infection (55). It is commonly known that L. lactis is widespread in the food industry and is generally recognized as safe (56). However, it is unexpected that L. lactis may also be an opportunistic bacterium for causing septicemia, cerebellar abscess, peritonitis, or endocarditis (57). Furthermore, L. garvieae can also cause sepsis, endocarditis, etc., and is well known as a potential zoonotic bacterium. This bacterium was detected (positive rate: 40%, Table S2) and cultured (100%, Fig. 3) in our bat fecal samples (58, 59), suggesting that we should increase discussion of *Lactococcus* spp. as potential pathogens, especially when they are detected in and cultured from wild animals, such as bats.

Some previous studies have shown that the type of hemolysis experiment performed in this study is related to four hemolysin genes (*tlyA*, *tlyB*, *tlyC*, and *hlyA*) and four putative hemolysin genes (hemolysin, hemolysin activation protein, hemolysin III, and hemolysin channel protein) found in bacteria (60, 61). However, there was no statistically significant correlation in this study between the hemolysin gene and hemolysis results (*P* > 0.05). Although some isolates caused hemolysis, hemolysin genes were not detected in the isolates, indicating that the hemolytic phenotype may be regulated by different genes and will require more research to be identified.

It is generally believed that the LDH cytotoxicity experiment is related to offensive virulence factors (such as adherence genes, toxin genes, and secretion system genes) in bacteria (62). Consistently, the numbers of adherence genes and secretion system genes were positively correlated with the cytotoxicity of the strains (*P* < 0.005). Unexpectedly, there was no correlation between the number of toxin genes and the cytotoxic results, which may be due to the lack of expression of relevant genes and warrants further research and elucidation. Significantly, the adherence genes were both proportional to the hemolysis and cytotoxicity results. Studies have shown that the adhesion ability of bacteria plays a critical early role in the pathogenesis of infectious diseases (63). These results indicate that the number of adhesion genes can be used as an area of focus for pathogenicity evaluation.

In summary, our findings indicated that bats carry numerous bacteria with pathogenic importance and that these include a variety of known and unknown pathogenic bacteria. Pathogen shedding by bats would result in the dissemination of those pathogens through feces since bats are widely distributed and prefer to inhabit eaves, woodlands, caves, and other places and therefore can easily come into contact with humans (64). Further investigations are warranted to identify the microbiota of bats in greater detail and their potential implications for human health.

## MATERIALS AND METHODS

### Fecal sample collection and identification of bat species.

Five genera of bats—*Rousettus*, *Taphozous*, *Hipposideros*, *Rhinolophus*, and *Myotis*—were sampled from 2011 to 2013 in the southern part of China (across four provinces and five regions) (Fig. S1). None of the bats showed any apparent clinical signs of illness according to their normal behavior and smooth fur appearance, and no dead bats were found in the caves (16). Collection, storage, and transportation of bat fecal samples and isolation of bacterial species were carried out as described in previous studies (65–67). In brief, fresh fecal samples were collected from bats using an aseptic plastic cloth which was placed under known natural bat roosting sites (usually in caves) during the afternoon. The next morning, the fecal samples were transferred from the plastic cloth into sterile tubes, and immediately placed into a constant temperature box with ice bags. The samples were transported to the laboratory and stored at −80°C until use. Fecal samples obtained from the same bat species were mixed together in the laboratory to minimize variations within the same species (16). Bat species were identified by amplifying the cytochrome b (*cytb*) gene with the primers L14724ag-F and H15915ag-R (16, 68) and then blasting the sequence within the National Center of Biotechnology Information (NCBI), and the species were determined by finding the species in the database with the highest homology to the sequence in the sample. To further verify the relationships of bat species, we generated a phylogenetic tree based on *cytb* genes using MEGA X (https://www.megasoftware.net/) with the neighbor-joining (NJ) algorithms (69). The robustness of the tree was evaluated based on 1,000 bootstrap replicates (70).

### DNA extraction from bat fecal samples.

Genomic DNA was extracted from the mixed bat fecal samples using the QIAamp Fast DNA Stool minikit (Qiagen, cat. no. 51604) according to the manufacturer’s instructions, and a bead-beating (zirconia/silica beads) and violent mechanical oscillation step was introduced to facilitate bacteria lysis and improve yield. Once DNA extraction was complete, the purity of the extracts was tested using NanoDrop (Thermo Fisher Scientific, USA).

### The V3-V4 region of 16S rRNA gene sequencing and data analysis.

Briefly, the variable V3-V4 region of the bacterial 16S rRNA genes was amplified and sequenced on an Illumina MiSeq instrument using paired-end 2 × 300 reads with barcode-indexed universal primers 341F (5′-CCT ACG GGN GGC WGC AG-3′) and 805R (5′-GAC TAC HVG GGT ATC TAA TCC-3′) at Tianjin Biochip Corporation, China (71, 72). The cycling conditions for amplifying V3-V4 were 94°C and 5 min for initial denaturation; then 25 cycles of 94°C for 30 s, 55°C for 30 s, and 72°C for 30 s; and finally 7 min of final elongation at 72°C. Real-Time Analysis (RTA) software was used to perform image analysis and base calling and to assign base-by-base quality scores (19). The forward and reverse reads were merged using FLASH (73). After joining the paired-end reads, the quality control phase retained sequences with a mean sequence quality score of >20 using FASTX-Toolkit v0.0.14 (http://hannonlab.cshl.edu/fastx_toolkit/). Potential chimeric sequences were screened out using VSEARCH v1.11.1. (74). Next, quality-checked sequence reads were clustered into operational taxonomic units (OTUs) at 98.7% sequence identity beforehand using USEARCH v9.2.64 (24). Finally, all OTUs were classified at the lowest possible taxonomic rank using QIIME based on the Greengenes reference database (https://greengenes.secondgenome.com) with the default parameters (75).

### Metataxonomic analysis of fecal microbiota at the species level.

Full-length 16S rRNA high-throughput sequencing was performed on a PacBio RS II platform at Tianjin Biochip Corporation (76). Near full-length 16S rRNA genes were amplified using 10 μL of total DNA as the template and the universal primer set 27F (5′-AGA GTT TGA TCC TGG CTC AG-3′) and 1492R (5′-GNT ACC TTG TTA CGA CTT-3′) with 16-nt barcodes tagged at the 5′ end (100 μL of 2× PCR buffer, 8 μL of forward primer [10 μM], 8 μL of reverse primer [10 μM], 2 μL of KODFX, and 72 μL of H_2_O). PCR was performed using *KODFX* DNA polymerase (TOYOBO), and the amplification parameters were as follows: initial denaturation for 2 min at 94°C; 25 cycles of 10 s at 98°C, 30 s at 52°C, and 1 min 30 s at 68°C; and finally a final elongation step of 10 min at 68°C. The primary sequences generated were processed through SMRT Portal (v2.3.0) provided by Pacific Biosciences (www.pacb.com/devnet/). To ensure that the barcoded reads were appropriately assigned to their original samples, a minimum barcode score of 22 was chosen to achieve an accuracy of 99.5%. Data containing ambiguous bases were deleted, primer sequences and adapters from the filtered reads were removed, and sequences outside nucleotide positions 10 to 1,490 were trimmed. The 16S cyclic consensus sequence was analyzed using standard tools in Mot package 2, UCHIME, and Arb (77, 78). Operational phylogenetic unit analyses were performed as previously described (76).

### Culturomics analysis.

Bacteria were isolated as described previously (67, 79). In brief, bat fecal samples collected from the same place were mixed and ground. Approximately 1 g of the ground fecal matter was placed in a sterile 1.5-mL EP centrifuge tube and mixed thoroughly with 1 mL of 0.85% normal saline. Then, 150 μL of this solution was transferred with a pipette and spread onto culture plates, including brain heart infusion (BHI) agar, BHI supplemented with 5.0% sheep blood agar, tryptone soy agar (TSA), R2A agar, and thiosulfate citrate bile salt sucrose agar. The uniformly coated medium was placed in an incubator for 7 days. Culture conditions were the result of the free combination of two temperatures (28°C and 37°C) and 3 gas conditions (aerobic, 5.0% CO_2_, and anaerobic [80.0% N_2_, 10% CO_2_, and 10% H_2_]; Table S1). Next, emerging colonies with different morphologies were selected, purified, and stored at −80°C in BHI broth supplemented with glycerol (20%, vol/vol) for further study.

The genomes were sequenced on an Illumina HiSeq 2000 platform with the respective extracted DNA of the bacterial pellet of the pure strains using the Wizard Genomic DNA purification kit (Promega), according to the manufacturer’s instructions, and assembled by SOAPdenovo (80). Gene calling and annotation were performed using the RAST (Rapid Annotation using Subsystem Technology) server (https://rast.nmpdr.org/) and the SEED viewer framework (81).

### Phylogenetic analyses.

The genomic DNA of each isolate was extracted and purified using a Wizard Genomic DNA purification kit (Promega). Pure culture strains were sequenced on the Illumina HiSeq 2000 platform and assembled using Velvet to obtain their respective draft genome sequences (82). The almost-complete 16S rRNA genes of strains were amplified and sequenced using the universal bacterial primers 27F and 1492R (67). The parameters for amplification were as follows: initial denaturation at 94°C for 5 min; followed by 30 cycles at 94°C for 30 s and 55°C for 30 s, and an extension step of 72°C for 90 s; with a final extension at 72°C for 10 min. The amplified sequences of our isolates were determined and compared with their corresponding sequences in the EzBioCloud database (https://www.ezbiocloud.net/identify) and GenBank by basic local alignment search tool (BLAST) search (https://blast.ncbi.nlm.nih.gov/Blast.cgi) to locate taxonomic positions. Phylogenetic trees were constructed using the NJ method (69) using MEGA X software (www.megasoftware.net) with a bootstrap analysis of 1,000 replications (70), and Kimura’s two-parameter method (83) was used to reconstruct NJ trees.

### Pathogen detection and virulence potential analysis.

A database containing 16S rRNA genes of well-identified bacterial pathogens was constructed from existing pathogen databases, including the National Microbial Pathogen Database Resource and Risk Group Database from the American Biological Safety Association (https://my.absa.org/Riskgroups). 16S rRNA genes in bacterial pathogens were retrieved from the Ribosomal Database Project (http://rdp.cme.msu.edu/index.jsp). Local BLAST was performed using our sequencing data (including the 16S rRNA gene sequences of the isolated strains) with the database and pathogen database as the query. To ensure maximum reliability, only results with sequence identity higher than 99% were recorded and the corresponding reports were searched in the PubMed database. For potential new bacterial species (16S rRNA gene sequence similarity <98.7%), phylogenetic trees were used to locate their taxonomic positions using the constructed NJ algorithms with the corresponding 16S rRNA gene sequences in the same pathogenic genus or family.

The virulence potential of isolated strains was analyzed by hemolytic analysis and cytotoxicity to cell lines. Three parallel controls were set for each experiment, and the experiment was repeated three times. The variation in assay results was statistically analyzed by the box plots, and the correlation between experimental results and genomic genes was shown with a correlation heatmap (https://www.chiplot.online/). For the bacterial hemolysis test, 8 mL of sterile defibrillated sheep blood was divided into two tubes and centrifuged at 1,000 × *g* for 10 min. Next, the supernatant was carefully pipetted out and discarded while taking care to avoid picking up red blood cells. Then, 1 mL of red blood cells was mixed with 49 mL of sterile phosphate-buffered saline to prepare 2% sheep red blood cells, and 150 μL of this mixture was then used to obtain 2-fold dilutions of the culture supernatant of experimental strains and the positive strain (Streptococcus suis). The microplate was incubated at 37°C (ambient air plus 5.0% CO_2_) for 2 h and centrifuged at 800 × *g* for 10 min. Then, 150 μL of the supernatant was transferred to a new plate and read at 540 nm with a microplate reader (BioTek Instruments, *Elx*808) (84).

The LDH cytotoxicity assay is a common method for determining cytotoxicity based on measuring the activity of cytoplasmic enzymes released by damaged cells. LDH is rapidly released into the cell culture supernatant when the plasma membrane is damaged, a key feature of cells undergoing apoptosis, necrosis, and other forms of cellular damage (85). Cytotoxicity was assessed using a Cytotox 96 Non-Radioactive Cytotoxicity assay kit (Promega, Madison, WI, USA) according to the manufacturer's instructions. Briefly, BV2 cells were purchased from the American Type Culture Collection (https://webstore.ansi.org/sdo/ATCC). BV2 cells (1 × 10^6^ cells/mL) were cultured for 2 days at 37°C with 5% CO_2_ in Dulbecco’s modified Eagle’s medium (DMEM) supplemented with 5% fetal bovine serum. Target strains were incubated with BV2 cells for 12 h at 37°C. LDH released from lysed target cells was measured in a microplate reader at 490 nm. To investigate the interaction between target strains and BV2 cells, cells were incubated with DMEM supplemented with 5% fetal bovine serum at 37°C for 12 h before the assay.

The hemolysis and cytotoxicity were calculated using the following formulas: hemolysis (%) = {[OD_540_ (optical density at 540 nm) sample − OD_540_ buffer]/[OD_540_ max − OD_540_ buffer]} × 100 and cytotoxicity (%) = (OD_490_ sample − OD_490_ negative control)/(OD_490_ max − OD_490_ buffer) × 100, calculated based on the average of replicates (86). Approximately 20% was regarded as the minimal hemolysis value compared to the positive control for hemolytic activity of FX-compounds (87), and because lactate dehydrogenase release over 20% has been used to evaluate the chemotherapy effect of advanced non-small cell lung cancer patients with platinum-based chemotherapy (88), 20% was listed as the cutoff for evaluating hemolysis and cytotoxicity in this study.

### Ethical approval.

The ethical practice was approved by Ethical Committee of the National Institute for Communicable Disease Control and Prevention, Chinese Center for Disease Control and Prevention (no. ICDC-2016004).

### Data availability.

The data sets generated for this study can be accessed from GenBank under accession no. PRJNA781098. The GenBank/EMBL/DDBJ accession numbers for the 16S rRNA gene sequences of potential novel species (culturomics) can be found in Fig. S6. The cytochrome b (*cytb*) gene sequences of B1-B5 were deposited into the GenBank database under accession numbers OP076971 to OP076975.
