# Supplementary material for: The Threat of Potentially Pathogenic Bacteria in the Feces of Bats
Source: Microbiol Spectr. 2022 Oct 26;10(6):e01802-22. doi: 10.1128/spectrum.01802-22 (PMC9769573; doi:10.1128/spectrum.01802-22)
Supplement: Supplemental file 1 — Tables S1-S5, Fig. S1-S11. Download spectrum.01802-22-s0001.pdf, PDF file, 3.6 MB [file spectrum.01802-22-s0001.pdf]

Journal name: *Microbiology Spectrum*

The threat of potentially pathogenic bacteria in the feces of bats

Yuyuan Huang[1#], Yamin Sun[2,3#], Qianni Huang[1,4], Xianglian Lv[1,5], Ji Pu[1], Wentao Zhu[1], Shan Lu[1,6], Dong Jin[1], Liyun Liu[1], Zhengli Shi[7], Jing Yang[1,6] and Jianguo Xu[1,2,6.8]*

1. State Key Laboratory of Infectious Disease Prevention and Control, National Institute for Communicable Disease Control and Prevention, Chinese Center for Disease Control and Prevention, Beijing 102206, PR China

2. Research Institute of Public Health, Nankai University, Tianjin 300350, PR China

3. Research Center for Functional Genomics and Biochip, Tianjin 300399, PR China

4. Guangxi Key Laboratory of AIDS Prevention and Treatment & Guangxi Collaborative Innovation Center for Biomedicine, School of Public Health, Guangxi Medical University, Nanning 530021, Guangxi, PR China

5. Department of Epidemiology, School of Public Health, Shanxi Medical University,

Taiyuan 030001, PR China

6. Research Units of Discovery of Unknown Bacteria and Function, Chinese Academy of Medical Sciences, Beijing 102206, PR China

7. CAS Key Laboratory of Special Pathogens and Biosafety, Wuhan Institute of Virology, Chinese Academy of Sciences, Wuhan 430071, PR China.

8. Peking University School of Public Health, Beijing 100083, PR China

[#]These authors contributed equally to this work

*Correspondence: Jianguo Xu, State Key Laboratory of Infectious Disease Prevention and Control, National Institute for Communicable Disease Control and Prevention, Chinese Center for Disease Control and Prevention, Changping, Beijing 102206, PR China. Email: xujianguo@icdc.cn; Tel: 8610 58790701; Fax: 8610 58790700.

Legends:

Table S1. Isolation and culture scheme of bat fecal samples.

Table S2. The OPUs annotation information.

Table S3. Species identified to be potential bacterial pathogens through metataxonomics from public databases.

Table S4. Species identified to be potential bacterial pathogens through culturomics from public databases.

Table S5. Hemolytic and cytotoxic genes analyses.

Figure S1. Sampling information of bats feces.

Figure S2. Phylogenetic dendrogram constructed from cytochrome b gene sequences of bats. Bar, 0.05.

Figure S3. Comparison of known species by Venn diagrams.

The following species were detected in culturomics and metataxonomics: *Advenella kashmirensis, Arthrobacter rhombi, Brachybacterium alimentarium, Brachybacterium fresconis, Citrobacter freundii, Enterobacter cancerogenus, Enterococcus gilvus, E. pallens, Fructilactobacillus ixorae, Hafnia psychrotolerans, Klebsiella michiganensis, K. pneumoniae, Lactococcus garvieae, L. lactis, Morganella morganii, Orbus sasakiae, Ornithinimicrobium murale, Pantoea agglomerans, Proteus cibarius, Pusillimonas noertemannii, Serratia glossinae, Weissella oryzae.*

Figure S4. Relative abundance bubble chart at the species level.

(A) Top 41 species detected in at least three samples; (B) Top 41 species with the highest abundance; The number indicates the quantity of 16S rRNA gene sequences; the same bright color represents the same species, and gray indicates other inconsistent species in A and B.

Figure S5. The multivariate correlation heatmap between metadata and nearly common microbiota of bats.

(A) Top 41 species detected in at least three samples; (B) Top 41 species with the highest abundance.

Figure S6. Phylogenetic tree based on near-complete 16S rRNA gene sequences revealed the position of potential novel species.

The red color indicates pathogenic bacteria, blue indicates the strains identified by metataxonomics, and green indicates the isolated and cultured strains.

Figure S7. Effect of culturing strains on sheep red blood cells (SRBC) and BV2 cells.

(A) Hemolysis of 2% sheep red blood cells; (B) cytotoxicity of BV2 challenged by the strains, BV2 treated with strains for 24 hours at 5×10^7 bacteria/plate.

Figure S8. The box plot of hemolytic (A) and cytotoxicity (B) assays variation.

Figure S9. Effects of the same strain on sheep red blood cells and BV2 cells.

(A) Hemolysis of 2% sheep red blood cells; (B) cytotoxicity of BV2 challenged by the strains, BV2 treated with strains for 24 hours at 5×10^7 bacteria/plate.

Figure S10. The multivariate correlation heatmap between hemolysis/cytotoxicity and related genes in sequenced genomes.

Figure S11. Isolation sources categories and oxygen demand of known species within metataxonomics (A) and culturomics (B).

The detailed information of the named species was obtained from the BacDive (https://bacdive.dsmz.de/) and LPSN (List of Prokaryotic names with Standing in Nomenclature, https://www.bacterio.net/species). The red branches indicate aerobic bacteria, purple indicates anaerobic bacteria, and earthy yellow indicates facultative anaerobic bacteria.

Table S1. Isolation and culture scheme of bat fecal samples.

Number	Medium	Gas conditions	Temperature (℃)
1	R2A	Aerobic	28
2	TSA		
3	BHI		
4	BHI-5% sheep blood		
5	TCBS		
6	R2A	Aerobic+5% CO2	
7	TSA		
8	BHI		
9	BHI-5% sheep blood		
10	TCBS		
11	R2A	Anaerobic (80.0% N_2, 10% CO_2 and 10% H_2)	
12	TSA		
13	BHI		
14	BHI-5% sheep blood		
15	TCBS		
16	R2A	Aerobic	37
17	TSA		
18	BHI		
19	BHI-5% sheep blood		
20	TCBS		
21	R2A	Aerobic+5% CO2	
22	TSA		
23	BHI		
24	BHI-5% sheep blood		
25	TCBS		
26	R2A	Anaerobic (80.0% N_2, 10% CO_2 and 10% H_2)	
27	TSA		
28	BHI		
29	BHI-5% sheep blood		
30	TCBS		

Table S2. The OPU annotation information.

OPU num	Taxon	B1	B2	B3	B4	B5
OPU 148	*Enterobacter cancerogenus*	10	233	11	573	75
OPU 169	*Klebsiella pneumoniae*	0	57	2	0	779
OPU 420	*Lactococcus lactis*	3	495	53	62	203
OPU 185	*Hafnia Obesumbacterium group**	12	0	436	145	54
OPU 278	*Advenella kashmirensis*	249	0	1	0	0
OPU 142	*Citrobacter freundii*	1	5	84	6	33
OPU 219	*Morganella morganii*	0	118	0	2	7
OPU 162	*Escherichia Shigella group**	80	0	0	0	46
OPU 194	*Gibbsiella dentisursi*	0	0	0	0	117
OPU 132	*Klebsiella aerogenes*	0	9	0	1	57
OPU 011	*Methylobacterium zarmanii*	16	7	21	0	5
OPU 149	*Enterobacter bugandensis*	0	6	4	0	32
OPU 199	*Serratia ficaria*	0	0	0	30	0
OPU 233	*Orbus sasakiae*	0	22	0	2	0
OPU 331	*Luteimonas soli*	21	0	0	1	1
OPU 181	*Cedecea davisae*	0	0	0	18	0
OPU 295	*Comamonas jiangduensis*	18	0	0	0	0
OPU 206	*Brenneria goodwinii*	0	0	0	0	14
OPU 167	*Citrobacter amalonaticus*	0	1	0	6	6
OPU 223	*Proteus cibarius*	0	0	1	2	10
OPU 213	*Pantoea agglomerans*	2	0	0	9	0
OPU 031	*Pannonibacter phragmitetus*	8	0	1	0	1
OPU 461	*Paraclostridium benzoelyticum*	0	0	7	0	3
OPU 190	*Serratia liquefaciens*	1	0	0	8	0
OPU 422	*Enterococcus gilvus*	0	5	0	2	1
OPU 211	*Kluyvera georgiana*	0	0	0	0	6
OPU 214	*Erwinia pericina*	0	0	0	6	0
OPU 372	*Haematomicrobium sanguinis*	4	0	2	0	0
OPU 419	*Lactococcus garvieae*	0	0	3	0	2
OPU 224	*Proteus mirabilis*	0	0	0	0	4
OPU 272	*Bordetella muralis*	4	0	0	0	0
OPU 273	*Bordetella tumulicala*	4	0	0	0	0
OPU 318	*Stenotrophomonas maltophilia group**	0	0	0	4	0
OPU 046	*Asaia bogorensis*	0	2	0	0	1
OPU 152	*Serratia nematodiphia*	1	1	1	0	0
OPU 182	*Klebsiella michiganensis*	0	2	0	1	0
OPU 212	*Pantoea ananatis*	1	0	0	1	1
OPU 255	*Acinetobacter dispersus*	0	0	0	3	0
OPU 415	*Weissella oryzae*	0	0	0	0	3
OPU 035	*Sphingomonas faeni*	2	0	0	0	0
OPU 092	*Sphingobacterium faecium*	1	1	0	0	0
OPU 168	*Salmonella enterica*	2	0	0	0	0
OPU 203	*Rouxiella chamberiensis*	0	0	0	2	0
OPU 215	*Erwinia iniecta*	1	0	0	1	0
OPU 243	*Halomonas muralis*	2	0	0	0	0
OPU 248	*Pseudomonas canadensis*	0	0	0	2	0
OPU 266	*Massilia aurea*	2	0	0	0	0
OPU 276	*Pusillimonas noertemannii*	2	0	0	0	0

OPU 294	*Xenophilus arseniciresistens*	2	0	0	0	0
OPU 304	*Pelomonas aquatica*	1	0	0	0	1
OPU 319	*Stenotrophomonas ginsengisoli*	2	0	0	0	0
OPU 320	*Stenotrophomonas terrae*	1	0	0	1	0
OPU 360	*Brachybacterium fresconis*	1	1	0	0	0
OPU 370	*Arthrobacter rhombi*	2	0	0	0	0
OPU 375	*Streptomyces coelescens group**	2	0	0	0	0
OPU 411	*Leuconostoc mesenteroides*	0	0	0	1	1
OPU 014	*Brevundimonas bullata*	1	0	0	0	0
OPU 015	*Brevundimonas naejiangsanensis*	0	0	1	0	0
OPU 021	*Rhizobium soli*	1	0	0	0	0
OPU 034	*Devosia riboflavina*	1	0	0	0	0
OPU 037	*Sphingomonas daechunensis*	1	0	0	0	0
OPU 068	*Myroides guanonis*	1	0	0	0	0
OPU 091	*Sphingobacterium nematocida*	1	0	0	0	0
OPU 140	*Citrobacter pasteuii*	0	0	1	0	0
OPU 209	*Tatumella punctata*	1	0	0	0	0
OPU 225	*Cosenzaea myxofaciens*	0	0	0	1	0
OPU 235	*Aeromonas rivipollensis*	1	0	0	0	0
OPU 236	*Aeromonas eucrenophila*	1	0	0	0	0
OPU 246	*Pseudomonas mohnii group**	0	0	0	1	0
OPU 249	*Pseudomonas protegens*	0	0	0	1	0
OPU 267	*Duganella zoogloeoides*	1	0	0	0	0
OPU 296	*Comamonas denitrificans*	0	0	0	0	1
OPU 300	*Delftia tsuruhatensis*	0	0	0	0	1
OPU 309	*Methylobacilus methanolivorans*	1	0	0	0	0
OPU 312	*Nitrosospira lacus*	1	0	0	0	0
OPU 322	*Pseudoxanthomonas yeongjuensis*	1	0	0	0	0
OPU 347	*Microbacterium gubbeenense*	1	0	0	0	0
OPU 353	*Janibacter cremeus*	1	0	0	0	0
OPU 354	*Ornithinimicrobium murale*	1	0	0	0	0
OPU 359	*Brachybacterium alimentarium*	1	0	0	0	0
OPU 366	*Brevibacterium linens*	0	1	0	0	0
OPU 369	*Glutamicibacter arilaitensis*	1	0	0	0	0
OPU 374	*Streptomyces flavovirens*	0	0	1	0	0
OPU 382	*Tsukamurella hongkongensis group**	0	0	1	0	0
OPU 383	*Dietzia timorensis*	1	0	0	0	0
OPU 393	*Bacillus drentensis*	1	0	0	0	0
OPU 408	*Staphylococcus equorum*	1	0	0	0	0
OPU 413	*Fructobacillus fructosus*	0	0	1	0	0
OPU 428	*Enterococcus phoeniculicola*	0	0	1	0	0
OPU_418	*Lactococcus sp1*	0	32	646	72	502
OPU_187	*Serratia sp5*	0	0	1	571	5
OPU_090	*Sphingobacterium sp2*	374	0	0	0	0
OPU_326	*Lysobacter sp1*	353	0	0	0	0
OPU_427	*Enterococcus sp5*	3	149	34	122	37
OPU_074	*Ulvibacter sp1*	0	0	257	0	0
OPU_139	*Citrobacter sp1*	0	89	0	90	48
OPU_287	*Castellaniella sp3*	0	0	165	0	1
OPU_263	*Salinisphaera sp1*	122	0	4	6	0

OPU_175	*Enterobacter sp11*	0	103	0	3	23
OPU_274	*Pusillimonas sp1*	105	0	3	0	1
OPU_075	*Gelidibacter sp1*	0	0	98	0	0
OPU_477	*Fusobacterium sp3*	0	0	0	86	0
OPU_452	*Clostridium sp3*	83	0	0	0	0
OPU_471	*Ureaplasma sp1*	45	3	12	0	22
OPU_405	*Pseudogracilibacillus sp2*	0	0	77	0	0
OPU_056	*Desulfatiferula sp1*	0	0	0	75	0
OPU_018	*Ochrobactrum sp1*	59	0	6	1	2
OPU_475	*Fusobacterium sp1*	0	0	0	7	58
OPU_241	*Halovibrio sp1*	60	0	0	0	0
OPU_192	*Serratia sp9*	0	0	0	59	0
OPU_227	*Providencia sp2*	0	17	4	28	10
OPU_242	*Halomonas sp1*	58	0	0	0	0
OPU_404	*Pseudogracilibacillus sp1*	0	0	58	0	0
OPU_123	*Arachidicoccus sp1*	37	0	17	0	0
OPU_338	*Luteibacter sp1*	31	0	8	14	0
OPU_002	*Rubellimicrobium sp1*	0	0	51	0	0
OPU_095	*Anseongella sp1*	51	0	0	0	0
OPU_455	*Candidatus Arthromitus sp1*	0	0	34	0	9
OPU_072	*Aequorivita sp3*	0	0	42	0	0
OPU_146	*Klebsiella sp4*	0	15	0	20	5
OPU_284	*Pusillimonas sp6*	0	0	38	0	0
OPU_017	*Bartonella sp1*	0	17	0	14	3
OPU_144	*Enterobacter sp1*	3	0	0	30	0
OPU_286	*Castellaniella sp2*	0	0	33	0	0
OPU_085	*Pedobacter sp4*	0	0	29	0	0
OPU_083	*Pedobacter sp2*	27	0	0	0	0
OPU_087	*Parapedobacter sp2*	27	0	0	0	0
OPU_340	*Luteibacter sp3*	2	0	25	0	0
OPU_424	*Enterococcus sp2*	0	26	0	0	0
OPU_279	*Eoetvoesia sp1*	7	0	18	0	0
OPU_321	*Pseudoxanthomonas sp1*	0	0	0	24	0
OPU_425	*Enterococcus sp3*	0	24	0	0	0
OPU_061	*Desulfovibrio sp2*	0	1	0	22	0
OPU_073	*Galbibacter sp1*	22	0	0	0	0
OPU_417	*Streptococcus sp1*	3	1	12	0	4
OPU_107	*Dysgonomonas sp1*	0	13	0	4	1
OPU_265	*Oxalobacter sp1*	0	0	0	18	0
OPU_100	*Apibacter sp1*	0	13	0	4	0
OPU_253	*Alcanivorax sp3*	0	0	16	0	0
OPU_257	*Acinetobacter sp3*	0	0	0	16	0
OPU_396	*Sporosarcina sp1*	0	0	16	0	0
OPU_410	*Lactobacillus sp1*	0	0	11	1	4
OPU_193	*Serratia sp6*	0	15	0	0	0
OPU_006	*Paracoccus sp3*	14	0	0	0	0
OPU_007	*Paracoccus sp4*	1	0	13	0	0
OPU_450	*Clostridium sp1*	0	0	1	7	6
OPU_060	*Desulfovibrio sp1*	0	4	0	9	0
OPU_070	*Aequorivita sp1*	0	0	13	0	0
OPU_397	*Sporosarcina sp2*	0	0	13	0	0

OPU_465	*Sporomusa sp1*	0	0	0	13	0
OPU_426	*Enterococcus sp4*	0	12	0	0	0
OPU_040	*Altererythrobacter sp2*	11	0	0	0	0
OPU_127	*Lewinella sp1*	0	0	11	0	0
OPU_166	*Pseudocitrobacter sp2*	0	0	0	8	3
OPU_176	*Enterobacter sp12*	0	9	0	0	2
OPU_238	*Vibrio sp1*	4	0	6	0	0
OPU_348	*Microbacterium sp4*	10	0	0	0	0
OPU_478	*Sebaldella sp1*	0	0	0	9	1
OPU_155	*Serratia sp4*	0	0	0	9	0
OPU_414	*Weissella sp1*	1	0	0	0	8
OPU_069	*Myroides sp3*	0	0	8	0	0
OPU_109	*Dysgonomonas sp3*	0	7	0	1	0
OPU_207	*Brenneria sp1*	0	0	0	0	8
OPU_283	*Pusillimonas sp5*	7	0	1	0	0
OPU_423	*Enterococcus sp1*	0	8	0	0	0
OPU_438	*Lachnoclostridium sp1*	0	0	0	6	2
OPU_088	*Pedobacter sp5*	7	0	0	0	0
OPU_131	*Truepera sp1*	1	0	6	0	0
OPU_153	*Serratia sp2*	0	2	1	2	2
OPU_178	*Enterobacter sp14*	0	4	0	1	2
OPU_200	*Serratia sp11*	0	0	0	7	0
OPU_218	*Erwinia sp4*	0	0	0	0	7
OPU_250	*Pseudomonas sp6*	0	6	0	1	0
OPU_259	*Acinetobacter sp5*	6	0	1	0	0
OPU_357	*Myceligenerans sp1*	7	0	0	0	0
OPU_165	*Pseudocitrobacter sp1*	0	0	0	0	6
OPU_198	*Gibbsiella sp5*	0	0	0	0	6
OPU_221	*Morganella sp3*	0	5	0	0	1
OPU_254	*Diplorickettsia sp1*	0	0	5	1	0
OPU_494	*Luteimonas sp*	6	0	0	0	0
OPU_001	*Roseovarius sp1*	0	0	5	0	0
OPU_013	*Microvirga sp1*	0	0	0	5	0
OPU_019	*Falsochrobactrum sp1*	0	0	5	0	0
OPU_048	*Kozakia sp1*	0	0	3	0	2
OPU_104	*Cryomorpha sp1*	0	0	5	0	0
OPU_110	*Bacteroides sp1*	0	3	0	2	0
OPU_151	*Enterobacter sp5*	0	4	0	1	0
OPU_154	*Serratia sp3*	0	3	0	0	2
OPU_157	*Enterobacter sp7*	0	0	0	5	0
OPU_261	*Acinetobacter sp7*	5	0	0	0	0
OPU_271	*Ignatzschineria sp1*	0	0	5	0	0
OPU_288	*Alcaligenes sp1*	0	0	5	0	0
OPU_332	*Luteimonas sp2*	5	0	0	0	0
OPU_351	*Leucobacter sp1*	0	0	5	0	0
OPU_390	*Ponticoccus sp1*	5	0	0	0	0
OPU_446	*Candidatus Soleaferrea sp2*	0	0	0	5	0
OPU_458	*Romboutsia sp1*	0	0	0	0	5
OPU_491	*Nitrospira sp1*	3	0	2	0	0
OPU_026	*Pseudaminobacter sp1*	4	0	0	0	0
OPU_093	*Sphingobacterium sp3*	3	0	1	0	0

OPU_101	*Alistipes sp1*	0	0	0	4	0
OPU_116	*Leadbetterella sp1*	0	0	0	0	4
OPU_126	*Taibaiella sp2*	0	0	4	0	0
OPU_197	*Gibbsiella sp4*	0	0	0	0	4
OPU_226	*Providencia sp1*	0	2	1	0	1
OPU_260	*Acinetobacter sp6*	3	0	0	0	1
OPU_277	*Candidimonas sp1*	3	0	1	0	0
OPU_282	*Pusillimonas sp4*	2	0	2	0	0
OPU_394	*Bacillus sp2*	4	0	0	0	0
OPU_429	*Enterococcus sp6*	0	2	0	2	0
OPU_445	*Candidatus Soleaferrea sp1*	0	0	0	4	0
OPU_453	*Clostridium sp4*	4	0	0	0	0
OPU_454	*Clostridium sp5*	0	0	0	0	4
OPU_472	*Mycoplasma sp2*	4	0	0	0	0
OPU_004	*Paracoccus sp1*	0	0	3	0	0
OPU_008	*Amaricoccus sp1*	3	0	0	0	0
OPU_028	*Mesorhizobium sp1*	2	0	1	0	0
OPU_042	*Novosphingobium sp1*	3	0	0	0	0
OPU_045	*Sandaracinobacter sp1*	0	0	3	0	0
OPU_047	*Heliconius sp1*	0	1	1	1	0
OPU_051	*Dongia sp2*	0	0	0	3	0
OPU_059	*Bradymonas sp1*	0	0	3	0	0
OPU_065	*Flavobacterium sp1*	3	0	0	0	0
OPU_105	*Parabacteroides sp1*	0	3	0	0	0
OPU_150	*Enterobacter sp4*	0	1	0	0	2
OPU_156	*Enterobacter sp6*	0	1	0	0	2
OPU_158	*Enterobacter sp8*	0	2	0	1	0
OPU_196	*Gibbsiella sp3*	0	0	0	0	3
OPU_210	*Kluyvera sp1*	0	2	0	1	0
OPU_222	*Morganella sp4*	0	2	0	0	1
OPU_230	*Plesiomonas sp1*	0	0	0	0	3
OPU_245	*Pseudomonas sp1*	3	0	0	0	0
OPU_258	*Acinetobacter sp4*	3	0	0	0	0
OPU_291	*Ottowia sp1*	1	0	0	1	1
OPU_310	*Methylotenera sp1*	3	0	0	0	0
OPU_329	*Lysobacter sp5*	3	0	0	0	0
OPU_333	*Dokdonella sp1*	3	0	0	0	0
OPU_342	*Rhodanobacter sp2*	0	0	2	1	0
OPU_350	*Agromyces sp2*	3	0	0	0	0
OPU_352	*Leucobacter sp2*	2	0	1	0	0
OPU_379	*Tomitella sp1*	0	0	3	0	0
OPU_460	*Paeniclostridium sp1*	0	0	1	0	2
OPU_005	*Paracoccus sp2*	0	0	2	0	0
OPU_012	*Methylobacterium sp2*	2	0	0	0	0
OPU_027	*Aquamicrobium sp2*	0	0	2	0	0
OPU_033	*Pelagibacterium sp1*	2	0	0	0	0
OPU_049	*Roseomonas sp1*	2	0	0	0	0
OPU_067	*Myroides sp2*	2	0	0	0	0
OPU_079	*Arenibacter sp1*	1	0	1	0	0
OPU_084	*Pedobacter sp3*	0	0	2	0	0
OPU_086	*Parapedobacter sp1*	2	0	0	0	0

OPU_089	*Sphingobacterium sp1*	0	0	2	0	0
OPU_102	*Alistipes sp2*	0	2	0	0	0
OPU_108	*Dysgonomonas sp2*	0	0	0	2	0
OPU_111	*Fermentimonas sp1*	0	0	1	0	1
OPU_121	*Terrimonas sp1*	2	0	0	0	0
OPU_122	*Ferruginibacter sp1*	1	0	1	0	0
OPU_134	*Klebsiella sp2*	0	0	0	0	2
OPU_135	*Klebsiella sp3*	0	0	0	1	1
OPU_138	*Raoultella sp1*	0	1	0	0	1
OPU_141	*Lelliottia sp1*	0	0	0	0	2
OPU_143	*Citrobacter sp4*	0	0	0	0	2
OPU_145	*Enterobacter sp2*	0	0	0	2	0
OPU_159	*Enterobacter sp9*	0	2	0	0	0
OPU_161	*Trabulsiella sp1*	0	0	1	0	1
OPU_179	*Enterobacter sp15*	0	1	0	1	0
OPU_184	*Shimwellia sp1*	0	0	0	1	1
OPU_195	*Gibbsiella sp2*	0	0	0	2	0
OPU_220	*Morganella sp2*	0	2	0	0	0
OPU_228	*Edwardsiella sp1*	0	0	1	1	0
OPU_229	*Arsenophonus sp1*	0	0	0	2	0
OPU_239	*Aliidiomarina sp1*	0	0	2	0	0
OPU_256	*Acinetobacter sp2*	0	0	0	0	2
OPU_289	*Paenalcaligenes sp1*	0	0	2	0	0
OPU_292	*Ottowia sp2*	1	0	0	0	1
OPU_297	*Comamonas sp3*	0	0	0	0	2
OPU_298	*Comamonas sp4*	2	0	0	0	0
OPU_305	*Rhizobacter sp1*	2	0	0	0	0
OPU_311	*Nitrosospira sp1*	2	0	0	0	0
OPU_323	*Luteimonas sp1*	2	0	0	0	0
OPU_324	*Luteimonas sp2*	2	0	0	0	0
OPU_328	*Lysobacter sp4*	1	0	1	0	0
OPU_335	*Arenimonas sp2*	2	0	0	0	0
OPU_336	*Pseudofulvimonas sp1*	2	0	0	0	0
OPU_337	*Rhodanobacter sp1*	1	0	0	1	0
OPU_339	*Luteibacter sp2*	0	0	2	0	0
OPU_345	*Microbacterium sp1*	1	0	1	0	0
OPU_346	*Microbacterium sp2*	2	0	0	0	0
OPU_355	*Promicromonospora sp1*	2	0	0	0	0
OPU_361	*Bogoriella sp1*	0	0	2	0	0
OPU_365	*Brevibacterium sp2*	2	0	0	0	0
OPU_367	*Brevibacterium sp3*	2	0	0	0	0
OPU_368	*Brevibacterium sp4*	2	0	0	0	0
OPU_376	*Streptomyces sp3*	2	0	0	0	0
OPU_380	*Rhodococcus sp1*	2	0	0	0	0
OPU_381	*Rhodococcus sp2*	2	0	0	0	0
OPU_385	*Nakamurella sp1*	0	0	2	0	0
OPU_400	*Kurthia sp1*	0	0	2	0	0
OPU_421	*Lactococcus sp1*	0	2	0	0	0
OPU_432	*Melissococcus sp1*	0	1	0	1	0
OPU_433	*Erysipelothrix sp1*	0	0	2	0	0
OPU_436	*Erysipelatoclostridium sp1*	0	1	0	1	0

OPU_470	*Mycoplasma sp1*	0	0	0	0	2
OPU_476	*Fusobacterium sp2*	2	0	0	0	0
OPU_486	*Tepidisphaera sp1*	0	0	2	0	0
OPU_010	*Methylobacterium sp1*	0	1	0	0	0
OPU_016	*Brevundimonas sp1*	1	0	0	0	0
OPU_020	*Paenochrobactrum sp1*	0	0	1	0	0
OPU_022	*Rhizobium sp2*	1	0	0	0	0
OPU_023	*Rhizobium sp3*	1	0	0	0	0
OPU_024	*Agrobacterium sp1*	1	0	0	0	0
OPU_025	*Aquamicrobium sp1*	0	0	1	0	0
OPU_029	*Nitratireductor sp1*	0	0	1	0	0
OPU_030	*Hoegflea sp1*	1	0	0	0	0
OPU_032	*Devosia sp1*	0	0	1	0	0
OPU_036	*Sphingomonas sp2*	1	0	0	0	0
OPU_038	*Sphingomonas sp4*	0	0	1	0	0
OPU_039	*Sphingobium sp1*	0	0	1	0	0
OPU_041	*Alterythrobacter sp1*	1	0	0	0	0
OPU_043	*Sphingopyxis sp1*	0	0	0	1	0
OPU_044	*Sphingorhabdus sp1*	1	0	0	0	0
OPU_050	*Dongia sp1*	1	0	0	0	0
OPU_052	*Constrictibacter sp1*	1	0	0	0	0
OPU_057	*Vulgatibacter sp1*	0	0	1	0	0
OPU_058	*Sandaracinus sp1*	1	0	0	0	0
OPU_062	*Desulfovibrio sp3*	0	0	0	1	0
OPU_066	*Myroides sp1*	1	0	0	0	0
OPU_071	*Aequorivita sp2*	0	0	1	0	0
OPU_080	*Arenibacter sp2*	0	0	1	0	0
OPU_082	*Pedobacter sp1*	1	0	0	0	0
OPU_097	*Chryseobacterium sp1*	1	0	0	0	0
OPU_099	*Moheibacter sp1*	0	0	1	0	0
OPU_112	*Porphyromonas sp1*	1	0	0	0	0
OPU_113	*Odoribacter sp1*	0	1	0	0	0
OPU_115	*Prevotella sp1*	1	0	0	0	0
OPU_117	*Hymenbobacter sp1*	1	0	0	0	0
OPU_118	*Hymenbobacter sp2*	1	0	0	0	0
OPU_119	*Hymenobacter sp3*	1	0	0	0	0
OPU_125	*Taibaiella sp1*	0	0	0	0	1
OPU_129	*Deinococcus sp1*	1	0	0	0	0
OPU_130	*Deinococcus sp2*	1	0	0	0	0
OPU_177	*Enterobacter sp13*	0	0	0	0	1
OPU_180	*Enterobacter sp16*	0	1	0	0	0
OPU_183	*Citrobacter sp1*	0	0	0	1	0
OPU_191	*Serratia sp8*	0	0	0	1	0
OPU_201	*Samsonia sp1*	1	0	0	0	0
OPU_202	*Leminorella sp1*	0	1	0	0	0
OPU_204	*Lonsdalea sp1*	0	0	0	0	1
OPU_208	*Buttiauxella sp1*	0	1	0	0	0
OPU_216	*Erwinia sp3*	0	0	0	1	0
OPU_217	*Pantoea sp1*	0	0	0	1	0
OPU_231	*Actinobacillus sp1*	1	0	0	0	0
OPU_232	*Actinobacillus sp2*	1	0	0	0	0

OPU_237	*Oceanisphaera sp1*	0	0	1	0	0
OPU_240	*Halomonas sp1*	1	0	0	0	0
OPU_247	*Pseudomonas sp3*	1	0	0	0	0
OPU_251	*Alcanivorax sp1*	1	0	0	0	0
OPU_252	*Alcanivorax sp2*	0	0	1	0	0
OPU_264	*Herminiimonas sp1*	0	0	1	0	0
OPU_268	*Massilia sp1*	1	0	0	0	0
OPU_269	*Massilia sp2*	1	0	0	0	0
OPU_270	*Lautropia sp1*	1	0	0	0	0
OPU_285	*Castellaniella sp1*	0	0	1	0	0
OPU_290	*Pseudorhodoferax sp1*	1	0	0	0	0
OPU_293	*Acidovorax sp1*	1	0	0	0	0
OPU_299	*Comamonas sp5*	1	0	0	0	0
OPU_301	*Melaminivora sp1*	0	0	0	0	1
OPU_302	*Lampropedia sp1*	1	0	0	0	0
OPU_303	*Aquincola sp1*	1	0	0	0	0
OPU_306	*Comamonas sp1*	1	0	0	0	0
OPU_308	*Methylovorus sp1*	0	0	1	0	0
OPU_325	*Lysobacter sp1*	1	0	0	0	0
OPU_330	*Lysobacter sp6*	0	0	0	1	0
OPU_334	*Chiayiivirga sp2*	1	0	0	0	0
OPU_341	*Oleiagrimonas sp1*	0	0	0	1	0
OPU_343	*Vicinamibacter sp1*	1	0	0	0	0
OPU_349	*Agromyces sp1*	1	0	0	0	0
OPU_356	*Isoptericola sp1*	1	0	0	0	0
OPU_358	*Brachybacterium sp1*	1	0	0	0	0
OPU_362	*Ruania sp1*	1	0	0	0	0
OPU_363	*Timonella sp1*	0	0	1	0	0
OPU_364	*Brevibacterium sp1*	0	1	0	0	0
OPU_371	*Acaricomes sp1*	1	0	0	0	0
OPU_373	*Nesterenkonia sp1*	1	0	0	0	0
OPU_377	*Mycobacterium sp1*	0	0	1	0	0
OPU_384	*Pseudonocardia sp1*	0	1	0	0	0
OPU_386	*Nocardiodes sp1*	1	0	0	0	0
OPU_388	*Microlunatus sp1*	1	0	0	0	0
OPU_389	*Microlunatus sp2*	0	1	0	0	0
OPU_395	*Bacillus sp3*	0	1	0	0	0
OPU_398	*Sporosarcina sp3*	0	0	0	0	1
OPU_399	*Bacillus sp4*	1	0	0	0	0
OPU_401	*Virgibacillus sp1*	1	0	0	0	0
OPU_402	*Oceanobacillus sp1*	1	0	0	0	0
OPU_403	*Oceanobacillus sp2*	1	0	0	0	0
OPU_409	*Staphylococcus sp1*	0	1	0	0	0
OPU_412	*Leuconostoc sp2*	0	0	0	1	0
OPU_416	*Weissella sp2*	1	0	0	0	0
OPU_430	*Enterococcus sp7*	1	0	0	0	0
OPU_431	*Vagococcus sp1*	0	0	0	1	0
OPU_434	*Gemella sp1*	1	0	0	0	0
OPU_440	*Dorea sp1*	0	1	0	0	0
OPU_442	*[Clostridium] sp1*	0	0	0	1	0
OPU_443	*[Clostridium] sp2*	0	0	0	1	0

OPU_447	*Acutalibacter sp1*	0	0	0	1	0
OPU_451	*Clostridium sp2*	0	0	1	0	0
OPU_459	*Eubacterium sp1*	0	0	1	0	0
OPU_462	*Anaerovorax sp1*	0	0	0	1	0
OPU_463	*Tissierella sp1*	0	0	0	0	1
OPU_464	*Hydrogenispora sp1*	0	1	0	0	0
OPU_473	*Mycoplasma sp3*	1	0	0	0	0
OPU_481	*Luteolibacter sp1*	0	0	1	0	0
OPU_482	*Prosthecobacter sp1*	1	0	0	0	0
OPU_483	*Pirellula sp1*	1	0	0	0	0
OPU_490	*Candidatus Tammella sp1*	0	1	0	0	0
OPU_495	*Morganella sp1*	0	0	0	0	1
OPU_497	*Comamonas sp*	0	0	0	0	1
OPU_499	*Hydrogenophaga sp*	1	0	0	0	0
OPU_500	*Leucobacter sp*	0	0	1	0	0
OPU_244	genus Oceanobacter	1	0	4	0	0
OPU_468	family Acidaminococcaceae	0	3	0	11	0
OPU_406	family Bacillaceae	2	1	14	0	0
OPU_128	family Balneolaceae	1	0	0	1	0
OPU_307	family Burkholderiaceae	0	0	0	2	0
OPU_124	family Chitinophagaceae	27	0	10	0	0
OPU_456	family Christensenellaceae	0	1	0	9	0
OPU_457	family Christensenellaceae	0	0	0	4	0
OPU_498	family Chromobacteriaceae	0	0	0	5	0
OPU_064	family Deferribacteraceae	0	2	0	0	0
OPU_063	family Desulfovibrionaceae	0	2	0	0	1
OPU_205	family Enterobacteriaceae	0	0	0	0	25
OPU_160	family Enterobacteriaceae	0	22	0	0	0
OPU_137	family Enterobacteriaceae	0	3	0	0	0
OPU_147	family Enterobacteriaceae	0	1	0	0	1
OPU_136	family Enterobacteriaceae	0	0	0	1	0
OPU_435	family Erysipelotrichaceae	0	10	0	1	0
OPU_081	family Flavobacteriaceae	127	0	456	0	0
OPU_076	family Flavobacteriaceae	290	0	1	0	0
OPU_098	family Flavobacteriaceae	1	0	0	0	0
OPU_054	family Helicobacteraceae	0	2	0	0	0
OPU_485	family Isosphaeraceae	0	0	2	0	0
OPU_441	family Lachnospiraceae	0	0	0	2	2
OPU_439	family Lachnospiraceae	0	0	1	2	0
OPU_391	family Lamiaceae	0	0	1	0	0
OPU_317	family Neisseriaceae	0	16	0	0	0
OPU_387	family Nocardioidaceae	3	0	0	0	0
OPU_234	family Orbaceae	0	1	0	0	2
OPU_493	family Orbaceae	0	0	0	1	0
OPU_437	family Paenibacillaceae	0	0	13	0	0
OPU_114	family Porphyromonadaceae	0	0	0	1	0
OPU_009	family Rhodobacteraceae	15	0	0	0	0
OPU_003	family Rhodobacteraceae	0	0	1	0	0
OPU_313	family Rhodocyclaceae	0	0	0	1	0
OPU_053	family Rickttsiaceae	1	0	0	0	0
OPU_103	family Rikenellaceae	0	0	0	0	1

OPU_444	family Ruminococcaceae	0	19	4	17	1
OPU_448	family Ruminococcaceae	0	0	0	6	0
OPU_449	family Ruminococcaceae	0	0	0	3	0
OPU_474	family Saccharimonadaceae	2	0	5	0	0
OPU_392	family Solirubrobacteraceae	0	0	1	0	0
OPU_094	family Sphingobacteriaceae	0	0	231	0	0
OPU_466	family Sporomusaceae	0	0	0	2	0
OPU_489	family Synergistaceae	0	0	1	13	0
OPU_106	family Tannerellaceae	0	1	0	2	0
OPU_469	family Veillonellaceae	10	0	0	0	0
OPU_467	family Sporomusaceae	0	0	0	3	1
OPU_055	class Deltaproteobacteria	0	0	0	6	1
OPU_262	class Gammaproteobacteria	2	0	0	0	0
OPU_314	class Betaproteobacteria	0	0	0	2	0
OPU_344	class Holophagae	0	0	1	0	0
OPU_492	class Gammaproteobacteria	0	0	0	1	0
OPU_496	class Rubrobacteridae	0	0	1	0	0
OPU_407	order Bacillales	1	1	0	0	0
OPU_316	order Betaproteobacteriales	6	0	0	0	0
OPU_315	order Betaproteobacteriales	2	0	0	0	0
OPU_479	order Chloroplast	153	1	0	3	0
OPU_120	order Cytophagales	3	0	0	0	0
OPU_480	order Gastranaerophilales	0	0	0	1	1
OPU_484	order Planctomycetales	2	0	0	0	0
OPU_487	phylum Planctomycetes	0	1	0	2	0

*, Reprensents taxon group which can't be distinguished well by 16S sequence;

B1, *Rousettus* spp.; B2, *Taphozous* spp.; B3, *Hipposideros* spp.; B4, *Rhinolophus* spp.; B5, Myotis spp.

Table S3. Species identified to be potential bacterial pathogens through metataxonomics from public databases.

Species	Clinic origins	Human Pathogen	Animal Pathogen	Plant Pathogen	Reference
Aeromonas eucrenophila	y				PMID: 15214640
Asaia bogorensis		y			PMID: 15264206
Brenneria goodwinii				y	PMID: 28570630
Cedecea davisae		y			PMID: 31579638
Citrobacter amalonaticus		y			Risk Group Database
Citrobacter freundii		y			Risk Group Database
Delftia tsuruhatensis		y			PMID: 29460754
Dietzia timorensis	y				PMID: 20220156
Enterobacter bugandensis		y			PMID: 29599516
Enterobacter cancerogenus		y		y	Risk Group Database
Enterococcus gilvus	y				PMID: 11923322
Erwinia persicina				y	PMID: 30781193
Escherichia Shigella group		y	y		Risk Group Database
Hafnia Obesumbacterium group		y			Risk Group Database
Klebsiella aerogenes		y			Risk Group Database
Klebsiella michiganensis		y			PMID: 30949345
Klebsiella pneumoniae		y	y		Risk Group Database
Kluyvera georgiana		y			Risk Group Database
Lactococcus garvieae		y	y		Risk Group Database
Methylobacterium zatmanii		y			PMID: 9854105
Morganella morganii		y	y		Risk Group Database
Pannonibacter phragmitetus		y			PMID: 33388179
Pantoea agglomerans		y			Risk Group Database
Pantoea ananatis				y	PMID: 27880983
Proteus mirabilis		y			Risk Group Database
Salmonella enterica		y			Risk Group Database
Serratia ficaria		y			PMID: 9774577
Serratia liquefaciens		y			Risk Group Database
Serratia nematodiphila			y		PMID: 25444874
Staphylococcus equorum	y	y			PMID: 23849315
Stenotrophomonas maltophilia		y			Risk Group Database
Tsukamurella hongkongensis		y			PMID: 29739926

Risk Group Database: https://my.absa.org/tiki-index.php?page=Riskgroups

Table S4. Species identified to be potential bacterial pathogens through culturomics from public databases

Species	clinic origins	Human Pathogen	Animal Pathogen	Plant Pathogen	Reference
Bacillus cereus		y	y		PMID: 18422617
Carnobacterium divergens		y			PMID: 25988484
Carnobacterium maltaromaticum			y		PMID: 22068485
Clostridium butyricum		y			PMID: 26493849
Enterococcus avium		y			PMID: 21667269
Enterococcus casseliflavus		y			PMID: 33158120
Enterococcus durans		y			PMID: 28590307
Enterococcus faecalis		y			PMID: 29923823
Enterococcus faecium		y			PMID: 29227922
Enterococcus gallinarum		y			PMID: 30931277
Enterococcus gilvus	y				PMID: 11923322
Enterococcus hirae		y			PMID: 31651255
Enterococcus pallens		y			PMID: 28348751
Lactococcus garvieae		y	y		PMID: 31010322
Leuconostoc pseudomesenteroides		y			PMID: 29434707
Staphylococcus aureus		y			PMID: 9709046
Staphylococcus caprae		y			PMID: 32315703
Staphylococcus cohnii		y			PMID: 33365133
Staphylococcus epidermidis		y			PMID: 26517189
Staphylococcus equorum	y				PMID: 16585638
Staphylococcus gallinarum		y			PMID: 31434672
Staphylococcus haemolyticus		y			PMID: 32799619
Staphylococcus hominis		y			PMID: 29355151
Staphylococcus lentus		y			PMID: 31072191
Staphylococcus nepalensis		y			PMID: 32818417
Staphylococcus saprophyticus		y			PMID: 29493989
Staphylococcus sciuri		y			PMID: 31769606
Staphylococcus succinus	y				PMID: 16585638
Staphylococcus xylosus			y		PMID: 31500280
Streptococcus sanguinis		y			PMID: 31347573
Virgibacillus marseillensis	y				PMID: 28280541
Weissella confusa		y			PMID: 21156010
Citrobacter murliniae			y		PMID: 14765902
Delftia lacustris		y			PMID: 25712727
Enterobacter cancerogenus		y			PMID: 9052388
Enterobacter huaxiensis	y				PMID: 30614784
Escherichia coli		y			PMID: 19966814
Escherichia fergusonii		y			PMID: 28616507
Hafnia alvei		y			PMID: 32025398

Klebsiella grimontii	y				PMID: 33888494
Klebsiella michiganensis		y			PMID: 30949345
Klebsiella pasteurii	y				PMID: 31708881
Klebsiella quasivariicola	y				PMID: 29051239
Klebsiella variicola		y			PMID: 31259664
Kluyvera intermedia	y				PMID: 28576740
Moraxella osloensis		y			PMID: 29409692
Morganella morganii		y			PMID: 32749130
Pantoea agglomerans		y		y	PMID: 27294620
Pantoea anthophila				y	PMID: 30699721
Raoultella ornithinolytica		y			PMID: 33818423
Serratia fonticola		y			PMID: 32528687
Serratia glossinae		y			PMID: 32268700
Serratia liquefaciens		y			PMID: 27466717
Serratia marcescens		y			PMID: 3519496
Brachybacterium paraconglomeratum		y			PMID: 32231828
Cellulomonas denverensis		y			PMID: 19656981
Cellulosimicrobium cellulans		y			PMID: 25786454
Dietzia aurantiaca	y				PMID: 21478395
Kocuria koreensis		y			PMID: 28138334
Kocuria marina		y			PMID: 28353459
Kocuria massiliensis		y			PMID: 29063496
Microbacterium paraoxydans		y			PMID: 30982728
Mycobacterium iranicum		y			PMID: 31723706
Mycobacterium mucogenicum		y			PMID: 19845703
Mycobacterium septicum		y			PMID: 32425103
Rhodococcus erythropolis		y			PMID: 22840370
Rhodococcus hoagii		y			PMID: 32154104
Chryseobacterium oranimense		y			PMID: 30640192
Chryseobacterium timonianum	y				PMID: 28509971

Table S5. Hemolytic and cytotoxic genes analyses.

Species	strains	Hemolysis (%)	Cytotoxicity (%)	Offensive virulence factors				Hemolysin genes				
				Adherence gene	Toxin gene	Secretion system gene	Sum	*tly*	Hemolysin	Hemolysin III	Hemolysin activation protein	Hemolysin channel protein
Bacillus altitudinis	HY1316	85	27.08	59	48	45	152	-	1	1	-	-
Serratia liquefaciens	HY590	81.1	76.8	87	50	60	197	-	1	1	-	-
Serratia fonticola	HY628	66	75.13	217	40	106	363	-	2	1	-	-
Serratia marcescens	HY1467	56	32.08	119	42	97	258	-	1	1	-	-
Enterococcus pallens	HY622	55.49	4.95	84	43	34	161	-	2		-	-
Bacillus siamensis	HY756	39	20.35	49	64	45	158	-	2	1	-	-
Weissella hellenica	HY604	32	13.37	29	20	13	62	-	1	1	-	-
Enterococcus casseliflavus	HY338	23	9.59	52	40	36	128	-	1	1	-	-
Enterococcus sp1.	HY326	22	3.76	66	56	42	164	-	1	1	-	-
Kocuria massiliensis	HY1904	20	7.65	17	27	31	75	-	1		-	-
Acidipropionibacterium jensenii	HY506	19.62	3.52	22	24	31	77	-	1		-	-
Acinetobacter sp.	HY1485	18.83	10.03	27	18	27	72	-	3	1	-	-
Aminobacter sp.	HY435	14.69	3.55	43	57	59	159	-	3		-	-
Apibacter raozihei	HY041	14.47	8.79	17	27	40	84	-	2	1	-	-
Arthrobacter sp.	HY1533	14.35	4.86	40	43	47	130	-	1		-	-
Arthrobacter luteolus	HY153	14.15	3.61	24	33	43	100	-	1		-	-
								-			-	-

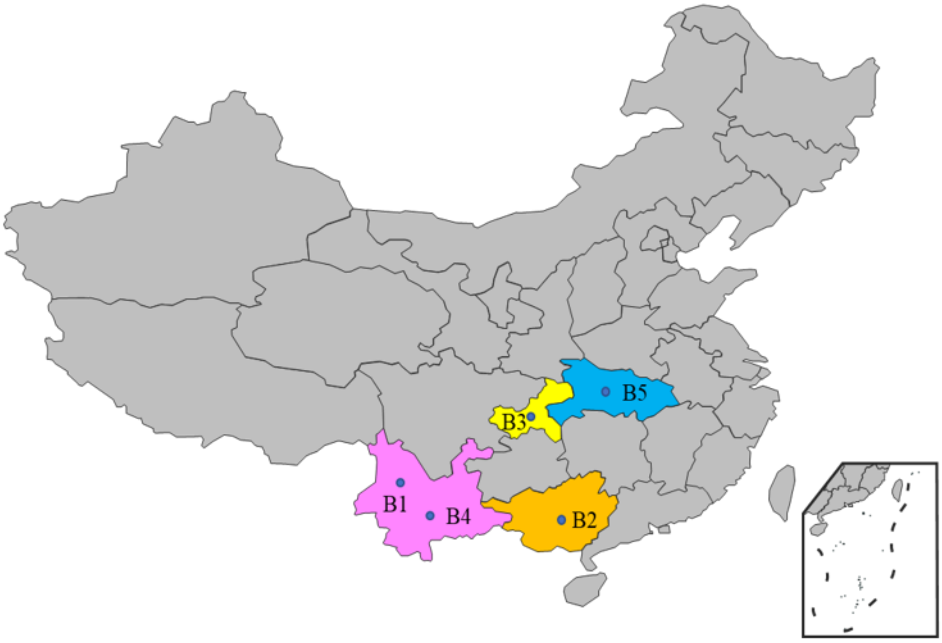

Samples	Genus	Feeding habit	Collecting time	Collecting location (Latitude and longitude)	Cytochrome b gene information			Cave information			
					Species	E value	Per. Ident	Temperature	Relative humidity	Altitude	Number of bats
B1	*Rousettus* spp.	Frugivorous	October 23, 2013	Chuxiong Yi Autonomous Prefecture, Yunnan Province (N25°09′10″, E102°04′39″)	*Rousettus leschenaultii*	0	97.19%	11.5°C	89.8%	1820 m	678
B2	*Taphozous* spp.	Insectivorous	July 28, 2011	Chongzuo City, Guangxi Province (N22°20′54″, E106°49′20″)	*Taphozous perforatus*	0	97.24%	30.1°C	61.4%	129 m	98
B3	*Hipposideros* spp.	Insectivorous	September 1, 2011	Changshou District, Chongqing City (N30°02′15″, E107°07′4″)	*Hipposideros cervinus*	0	95.74%	32.3°C	27.8%	416 m	112
B4	*Rhinolophus* spp.	Insectivorous	July 22, 2013	Jinning District, Kunming City, Yunnan Province (N24°33′58″, E102°25′57″)	*Rhinolophus macrotis*	0	96.67%	20.4°C	73.2%	1895 m	101
B5	*Myotis* spp.	Insectivorous	September 14, 2013	Xian'an District, Xianning City, Hubei Province (N29°46′56″, E114°18′13″)	*Myotis scotti*	0	95.09%	26.8°C	72.3%	114 m	142

Figure S1. Sampling information of bats feces.

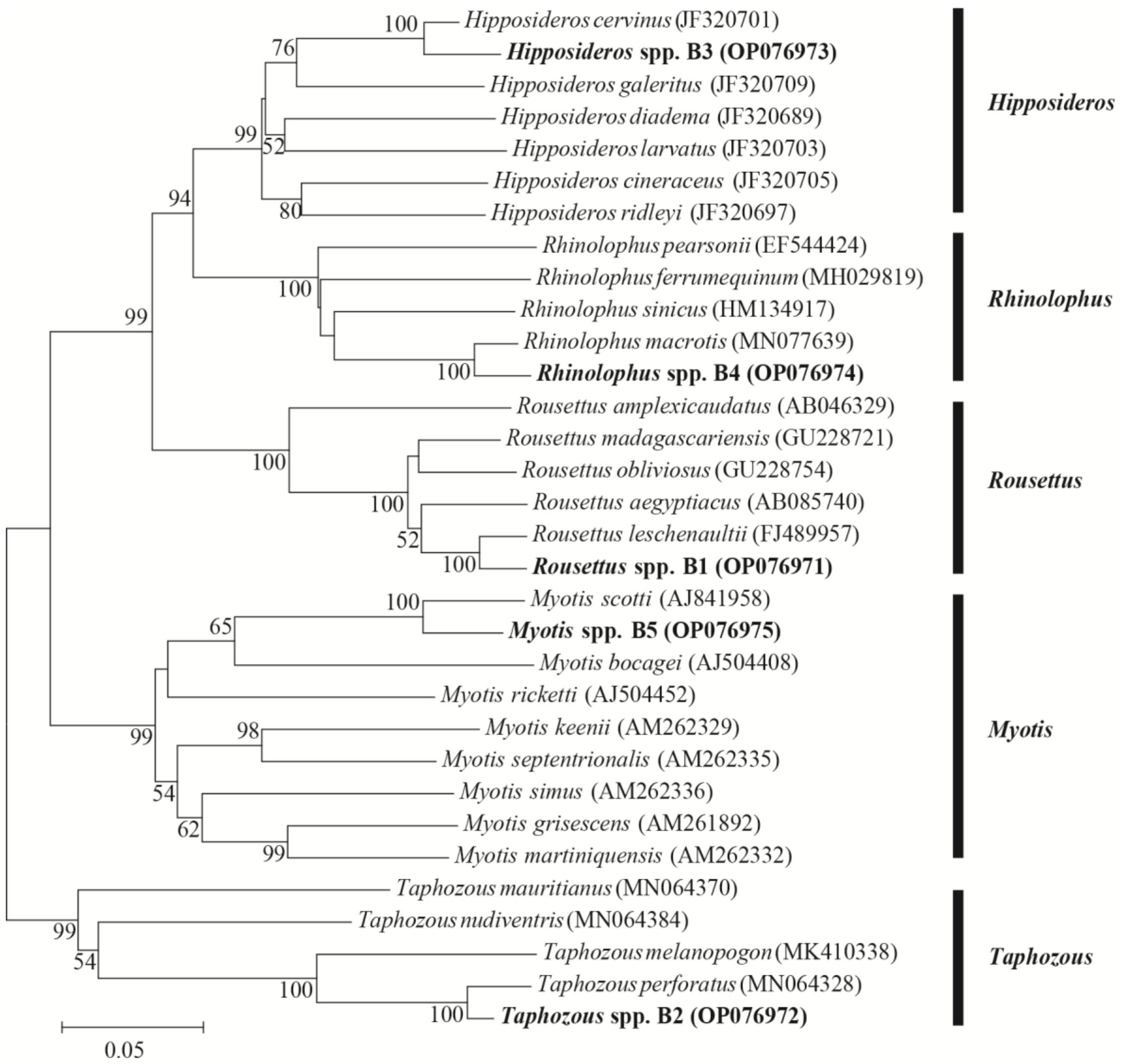

Figure S2. Phylogenetic dendrogram constructed from cytochrome b gene sequences of bats. Bar, 0.05.

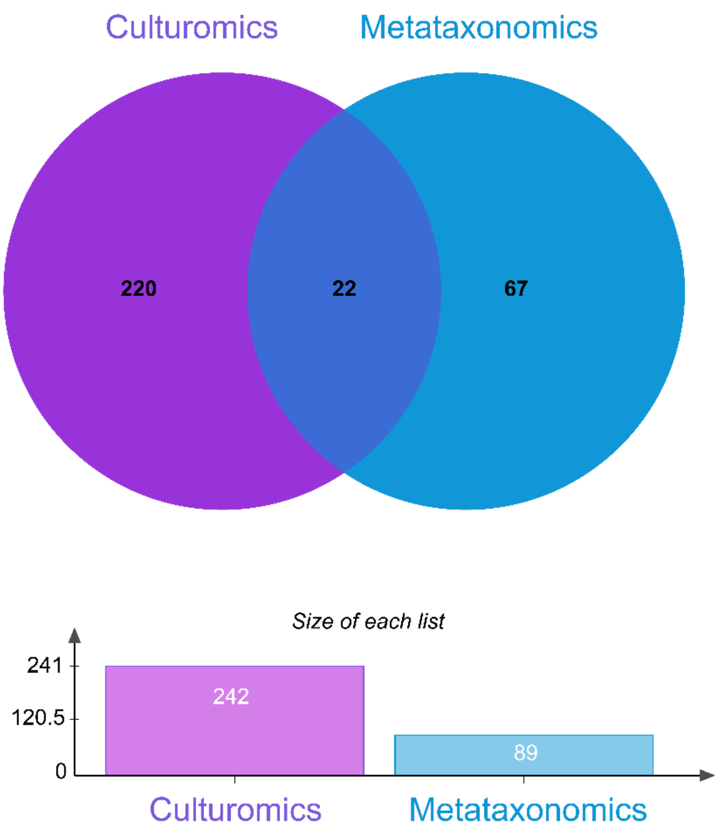

Figure S3. Comparison of known species by Venn diagrams.

The following species were detected in culturomics and metataxonomics: *Advenella kashmirensis, Arthrobacter rhombi, Brachybacterium alimentarium, Brachybacterium fresconis, Citrobacter freundii, Enterobacter cancerogenus, Enterococcus gilvus, E. pallens, Fructilactobacillus ixorae, Hafnia psychrotolerans, Klebsiella michiganensis, K. pneumoniae, Lactococcus garvieae, L. lactis, Morganella morganii, Orbus sasakiae, Ornithinimicrobium murale, Pantoea agglomerans, Proteus cibarius, Pusillimonas noertemannii, Serratia glossinae, Weissella oryzae.*

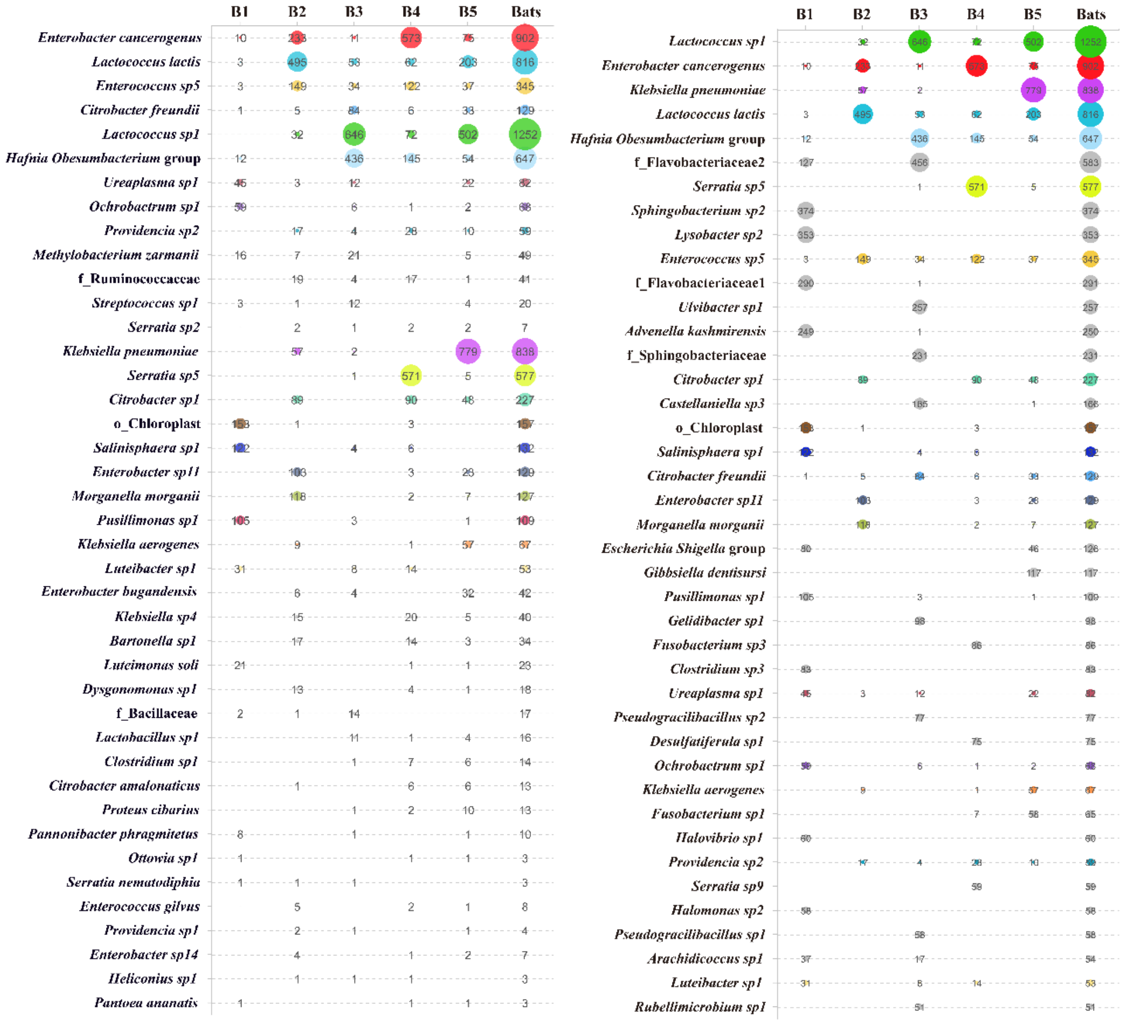

Figure S4. Relative abundance bubble chart at the species level.

(A) Top 41 species detected in at least three samples; (B) Top 41 species with the highest abundance. The number indicates the quantity of 16S rRNA gene sequences; the same bright color represents the same species, and gray indicates other inconsistent species in A and B.

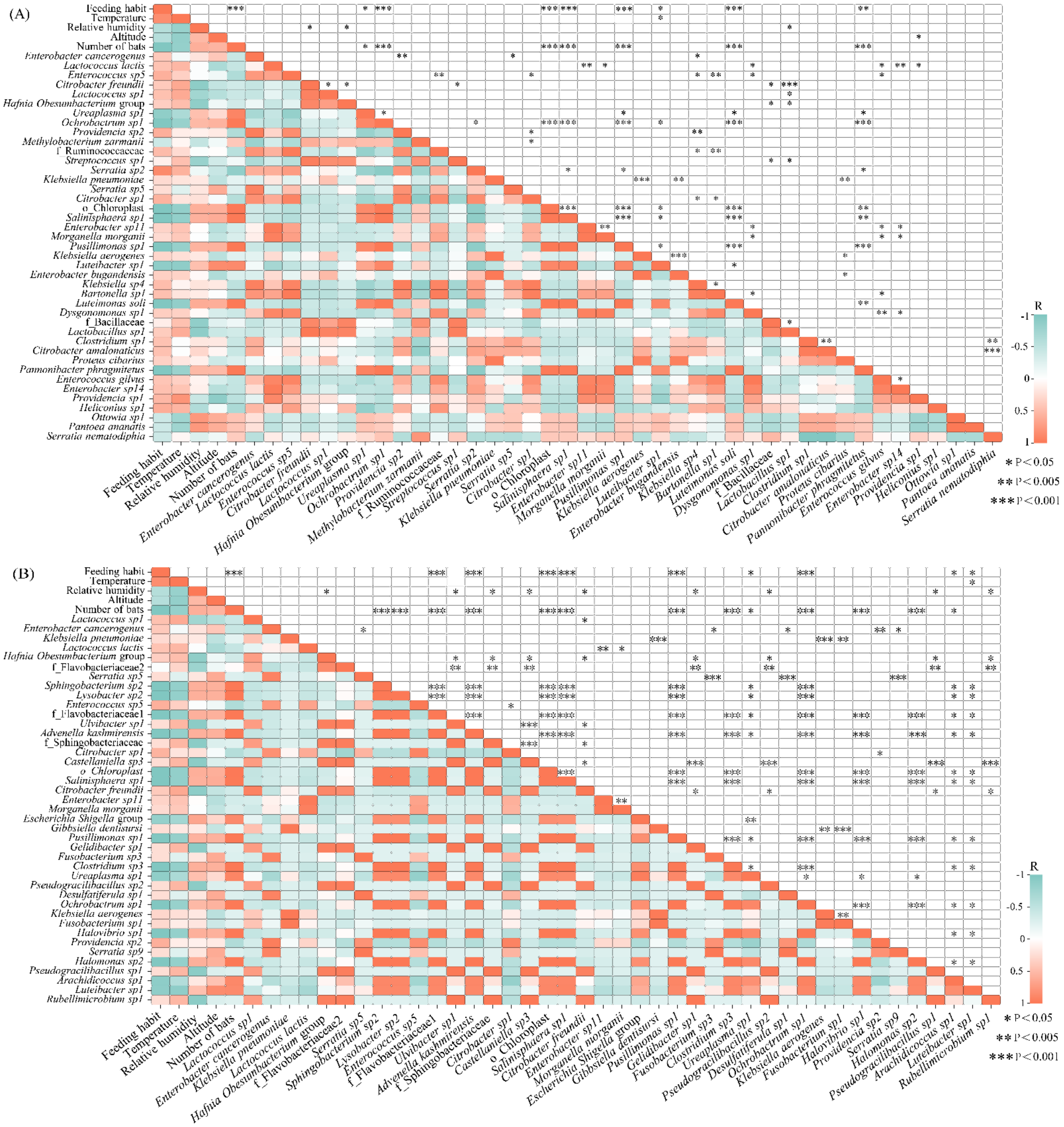

Figure S5. The multivariate correlation heatmap between metadata and nearly common microbiota of bats.

(A) Top 41 species detected in at least three samples; (B) Top 41 species with the highest abundance.

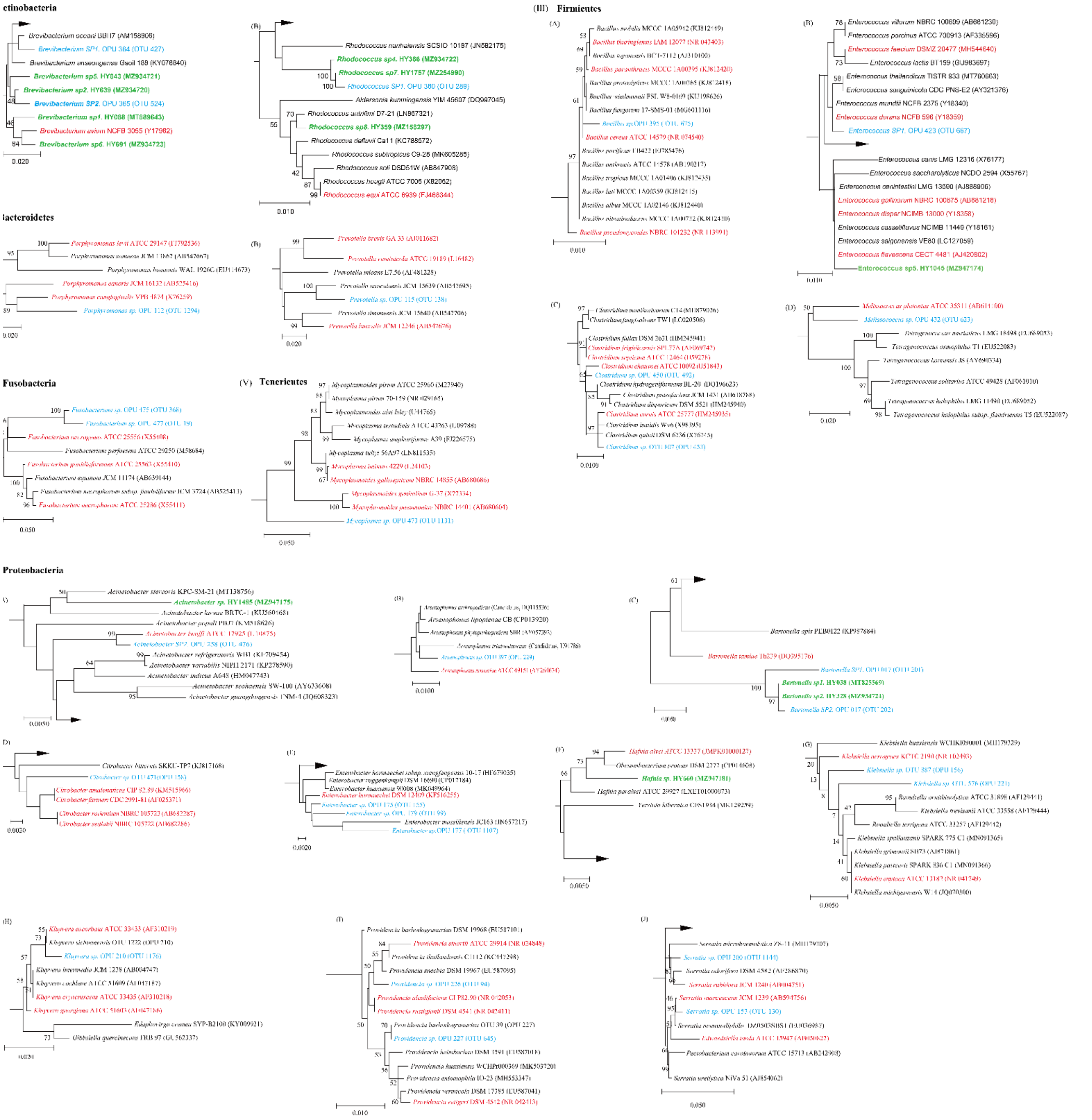

Figure S6. Phylogenetic tree based on near-complete 16S rRNA gene sequences revealed the position of potential novel species.

The red color indicates pathogenic bacteria, blue indicates the strains identified by metataxonomics, and green indicates the isolated and cultured strains.

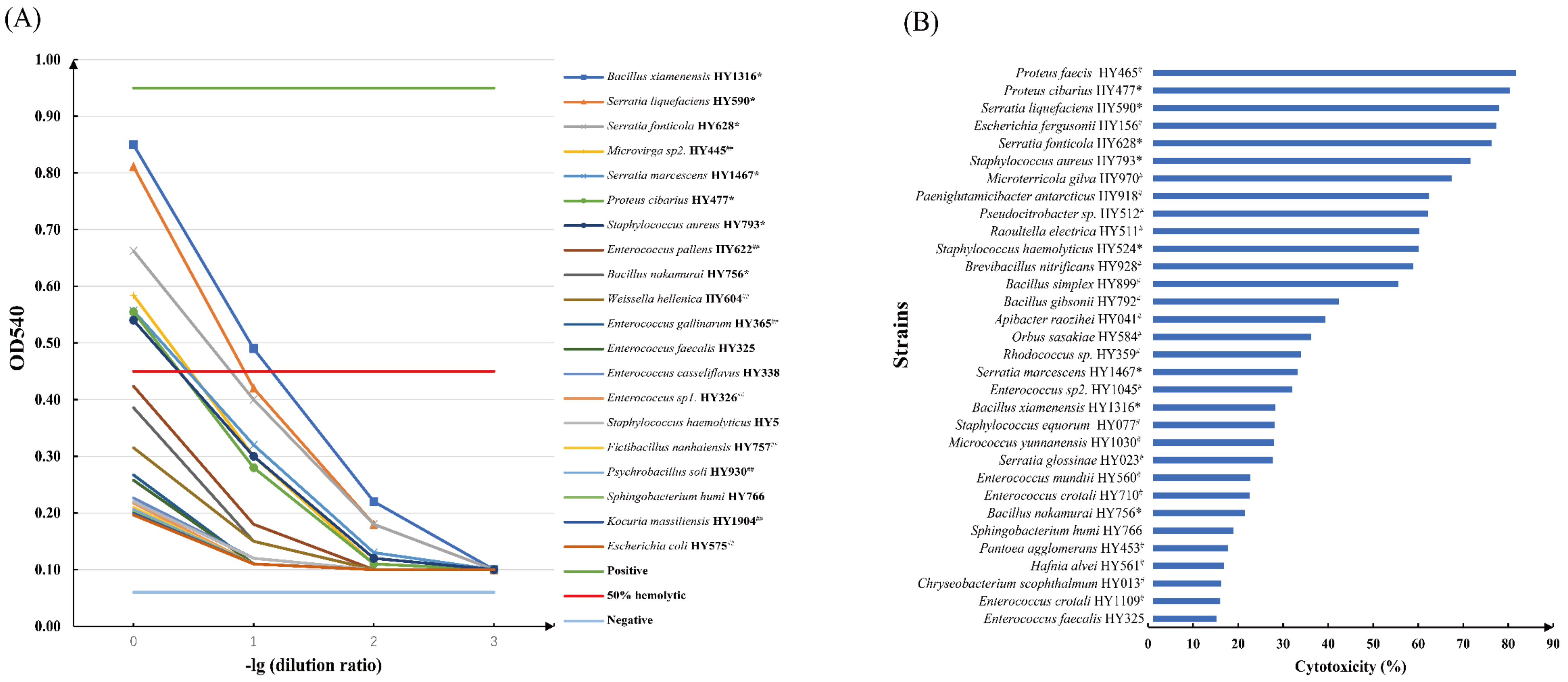

Figure S7. Effect of culturing strains on sheep red blood cells (SRBC) and BV2 cells.

(A) Hemolysis of 2% sheep red blood cells; (B) cytotoxicity of BV2 challenged by the strains, BV2 treated with strains for 24 hours at 5 × 10^7 bacteria/plate.

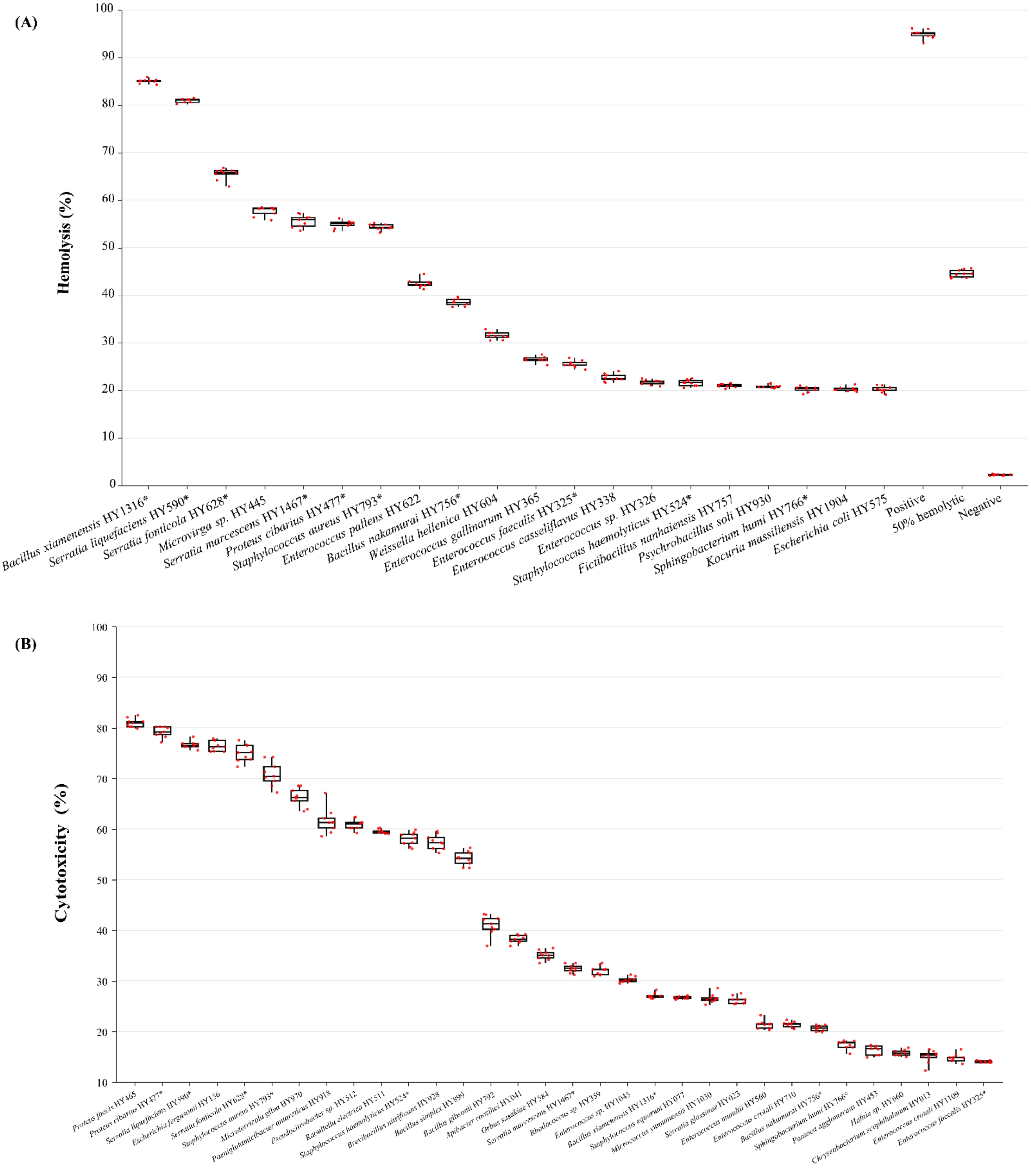

Figure S8. The box plot of hemolytic (A) **and cytotoxicity** (B) **assays variation**.

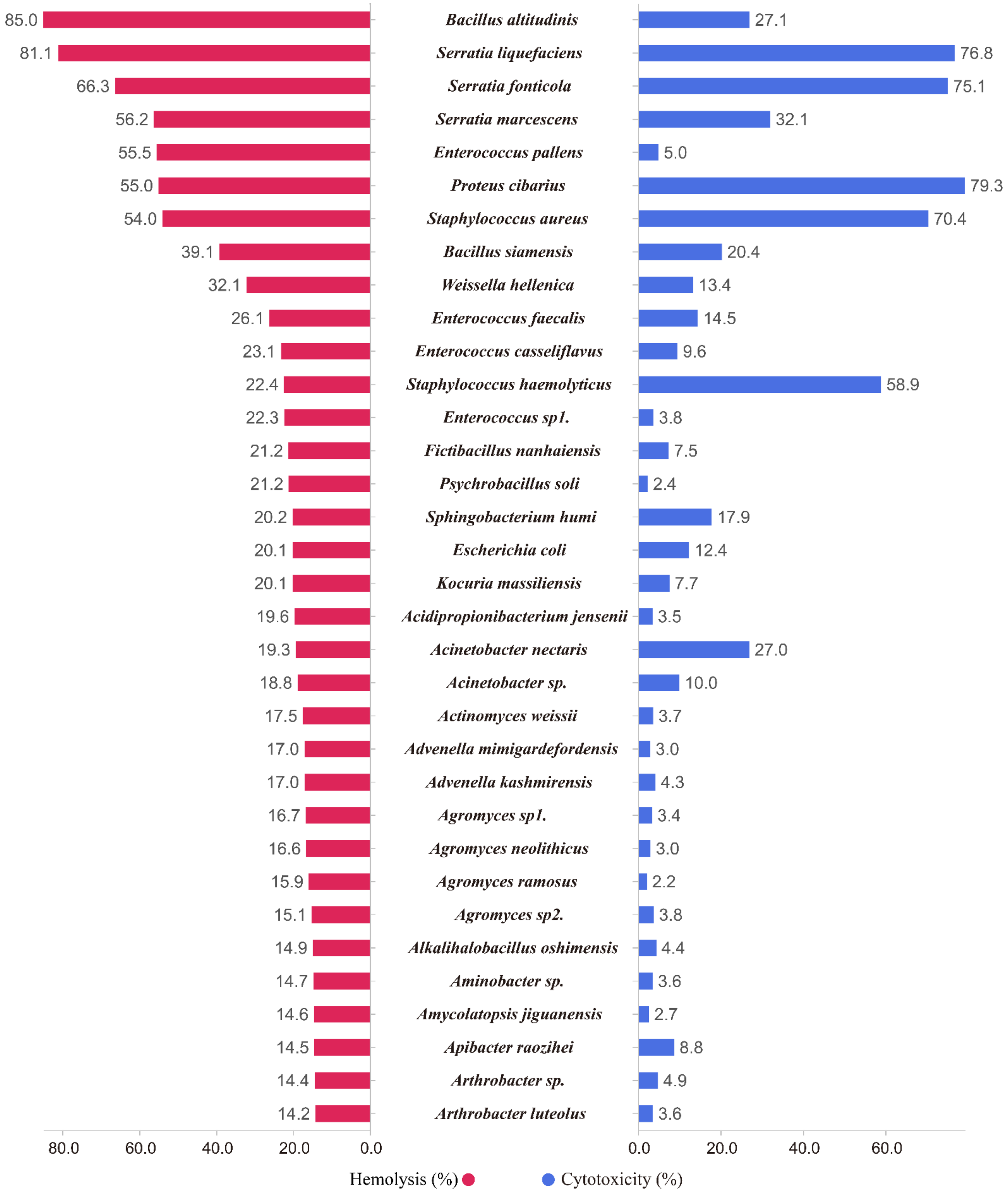

Figure S9. Effects of the same strain on sheep red blood cells and BV2 cells.

(A) Hemolysis of 2% sheep red blood cells; (B) cytotoxicity of BV2 challenged by the strains, BV2 treated with strains for 24 hours at 5 × 10^7 bacteria/plate.

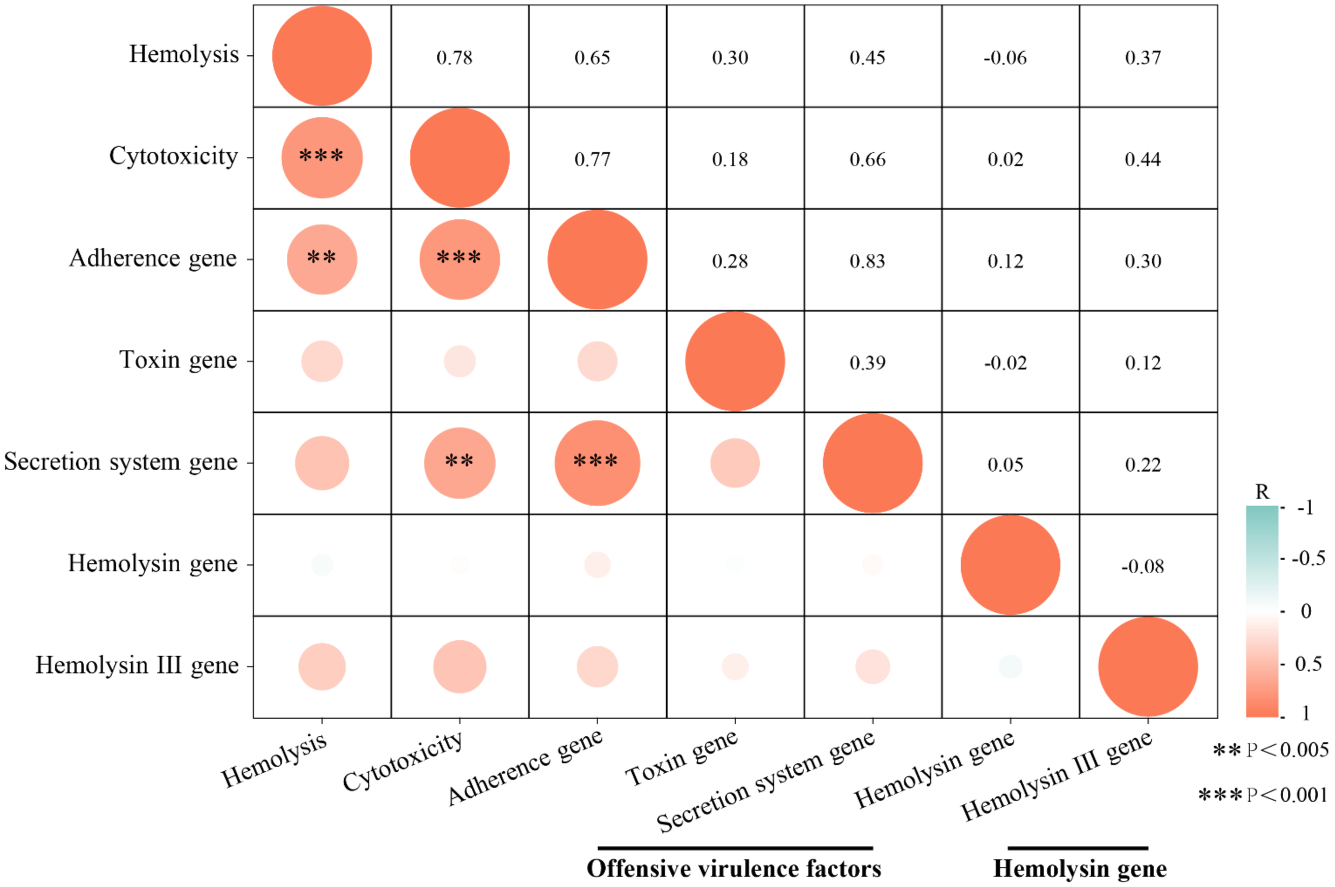

Figure S10. The multivariate correlation heatmap between hemolysis/cytotoxicity and related genes in sequenced genomes.

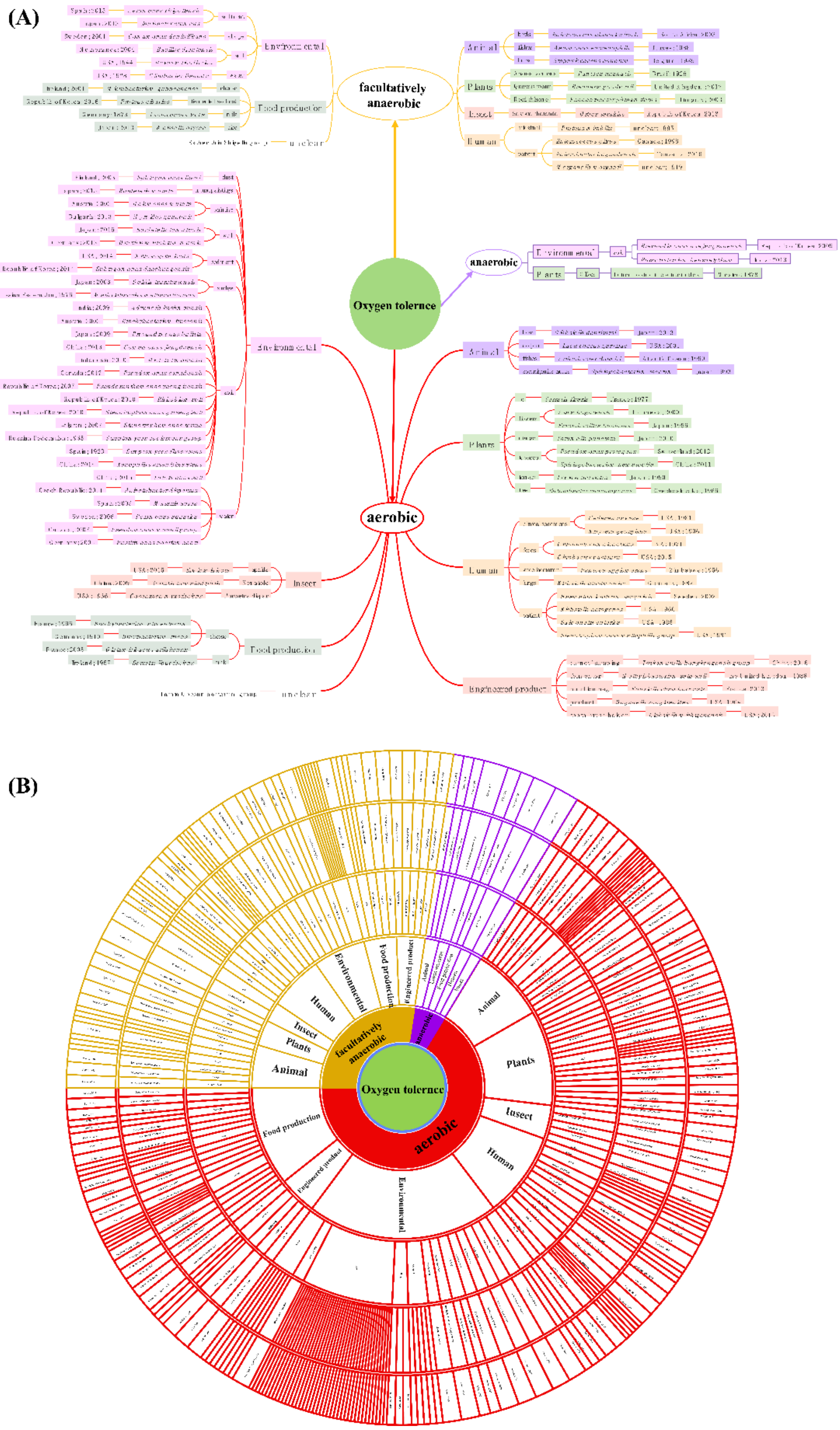

Figure S11. Isolation sources categories and oxygen demand of known species within metataxonomics (A) and culturomics (B).

The detailed information of the named species was obtained from the BacDive (https://bacdive.dsmz.de/) and LPSN (List of Prokaryotic names with Standing in Nomenclature, https://www.bacterio.net/species). The red branches indicate aerobic bacteria, purple indicates anaerobic bacteria, and earthy yellow indicates facultative anaerobic bacteria.
